# Neuronal α‐Synuclein Disease Stage Progression over 5 Years

**DOI:** 10.1002/mds.30191

**Published:** 2025-04-30

**Authors:** Tanya Simuni, Caroline Gochanour, Anuprita R. Nair, Michael C. Brumm, Christopher Coffey, Kathleen L. Poston, Lana M. Chahine, Daniel Weintraub, Caroline M. Tanner, Paulina Gonzalez‐Latapi, Catherine M. Kopil, Yuge Xiao, Sohini Chowdhury, Tien Dam, Gennaro Pagano, Diane Stephenson, Andrew Siderowf, Billy Dunn, Kenneth Marek, the NSD Working Group the Parkinson's Progression Markers Initiative, Kenneth Marek, Caroline Tanner, Tanya Simuni, Andrew Siderowf, Douglas Galasko, Lana Chahine, Christopher Coffey, Kalpana Merchant, Kathleen Poston, Roseanne Dobkin, Tatiana Foroud, Brit Mollenhauer, Dan Weintraub, Ethan Brown, Karl Kieburtz, Mark Frasier, Todd Sherer, Sohini Chowdhury, Roy Alcalay, Aleksandar Videnovic, Duygu Tosun‐Turgut, Werner Poewe, Susan Bressman, Jan Hammer, Raymond James, Ekemini Riley, John Seibyl, Leslie Shaw, David Standaert, Sneha Mantri, Nabila Dahodwala, Michael Schwarzschild, Connie Marras, Hubert Fernandez, Ira Shoulson, Helen Rowbotham, Paola Casalin, Claudia Trenkwalder, Todd Sherer, Sohini Chowdhury, Mark Frasier, Jamie Eberling, Katie Kopil, Alyssa O'Grady, Maggie McGuire Kuhl, Leslie Kirsch, Tawny Willson, Emily Flagg, Tanya Simuni, Bridget McMahon, Craig Stanley, Kim Fabrizio, Dixie Ecklund, Trevis Huff, Tatiana Foroud, Laura Heathers, Christopher Hobbick, Gena Antonopoulos, John Seibyl, Kathleen Poston, Christopher Coffey, Chelsea Caspell‐Garcia, Michael Brumm, Brit Mollenhauer, Doug Galasko, Kalpana Merchant, Andrew Singleton, Tatiana Foroud, Thomas Montine, Caroline Tanner, Carlie Tanner, Ethan Brown, Lana Chahine, Roseann Dobkin, Monica Korell, Charles Adler, Roy Alcalay, Amy Amara, Paolo Barone, Bastiaan Bloem Susan Bressman, Kathrin Brockmann, Norbert Brüggemann, Lana Chahine, Kelvin Chou, Nabila Dahodwala, Alberto Espay, Stewart Factor, Hubert Fernandez, Michelle Fullard, Douglas Galasko, Robert Hauser, Penelope Hogarth, Shu‐Ching Hu, Michele Hu, Stuart Isaacson, Christine Klein, Rejko Krueger, Mark Lew, Zoltan Mari, Connie Marras, Maria Jose Martí, Nikolaus McFarland, Tiago Mestre, Brit Mollenhauer, Emile Moukheiber, Alastair Noyce, Wolfgang Oertel, Njideka Okubadejo, Sarah O'Shea, Rajesh Pahwa, Nicola Pavese, Werner Poewe, Ron Postuma, Giulietta Riboldi, Lauren Ruffrage, Javier Ruiz Martinez, David Russell, Marie H. Saint‐Hilaire, Neil Santos, Wesley Schlett, Ruth Schneider, Holly Shill, David Shprecher, Tanya Simuni, David Standaert, Leonidas Stefanis, Yen Tai, Caroline Tanner, Arjun Tarakad, Eduardo Tolosa, Aleksandar Videnovic, Susan Ainscough, Courtney Blair, Erica Botting, Isabella Chung, Kelly Clark, Ioana Croitoru, Kelly DeLano, Iris Egner, Fahrial Esha, May Eshel, Frank Ferrari, Victoria Kate Foster, Alicia Garrido, Madita Grümmer, Bethzaida Herrera, Ella Hilt, Chloe Huntzinger, Raymond James, Farah Kausar, Christos Koros, Yara Krasowski, Dustin Le, Ying Liu, Taina M. Marques, Helen Mejia Santana, Sherri Mosovsky, Jennifer Mule, Philip Ng, Lauren O'Brien, Abiola Ogunleye, Oluwadamilola Ojo, Obi Onyinanya, Lisbeth Pennente, Romina Perrotti, Michael Pileggi, Ashwini Ramachandran, Deborah Raymond, Jamil Razzaque, Shawna Reddie, Kori Ribb, Kyle Rizer, Janelle Rodriguez, Stephanie Roman, Clarissa Sanchez, Cristina Simonet, Anisha Singh, Elisabeth Sittig, Barbara Sommerfeld, Angela Stovall, Bobbie Stubbeman, Alejandra Valenzuela, Catherine Wandell, Diana Willeke, Karen Williams, Dilinuer Wubuli

**Affiliations:** ^1^ Department of Neurology Northwestern University Feinberg School of Medicine Chicago Illinois USA; ^2^ Department of Biostatistics College of Public Health, University of Iowa Iowa City Iowa USA; ^3^ Department of Neurology Stanford University School of Medicine Palo Alto California USA; ^4^ Department of Neurology University of Pittsburgh Pittsburgh Pennsylvania USA; ^5^ Department of Psychiatry University of Pennsylvania and the Parkinson's Disease and Mental Illness Research, Education and Clinical Centers (PADRECC and MIRECC), Philadelphia Veterans Affairs Medical Center Philadelphia USA; ^6^ Department of Neurology, Movement Disorders and Neuromodulation Center Weill Institute for Neuroscience, University of California San Francisco California USA; ^7^ The Michael J. Fox Foundation for Parkinson's Research New York New York USA; ^8^ Neumora Boston Massachusetts USA; ^9^ F. Hoffmann‐La Roche Ltd. Basel Switzerland; ^10^ Critical Path for Parkinson's, Critical Path Institute Tucson Arizona USA; ^11^ Department of Neurology Perelman School of Medicine, University of Pennsylvania Philadelphia Pennsylvania USA; ^12^ Institute for Neurodegenerative Disorders New Haven Connecticut USA

**Keywords:** biological definition, neuronal α‐synuclein disease, Parkinson's disease, dementia with Lewy bodies

## Abstract

**Background:**

Neuronal α‐synuclein disease (NSD) is defined by the presence of an in vivo biomarker of neuronal alpha‐synuclein (n‐asyn) pathology. The NSD integrated staging system (NSD‐ISS) for research describes progression across the disease continuum as stages 0 to 6.

**Objective:**

The aim was to assess 5‐year longitudinal change in NSD‐ISS in early disease.

**Methods:**

Analysis included a subset of participants from the Parkinson's Progression Markers Initiative (PPMI) enrolled before 2020 as Parkinson's disease (PD) patients, prodromal PD patients, or healthy controls (HC) who met NSD criteria. Staging was defined based on biomarkers of n‐asyn and dopaminergic dysfunction in early stages, clinical features, and severity of functional impairment in stages 3 to 6. Stages were determined annually for 5 years.

**Results:**

Of 576 NSD participants, 494 were enrolled as PD patients, 74 prodromal PD patients, and 8 HCs. At baseline, 24% of participants were stage 2B, 56% Stage 3, 13% stage 4, and less than 5% in other stages. At year 5, the respective percentages for stages 2B to 4 were 11%, 50%, and 34%, indicating progression through NSD stages. Progression was driven by functional impairment in the predominantly motor domain (95%) for stage 2B to 3, increasing degree of nonmotor dysfunction for stages 3 to 4 (46%), and a combination of domains for stages 4 to 5. Initiation of dopaminergic medications led to stage regression in 8% of participants in Stage 3 but 41% in stage 4.

**Conclusions:**

Our analysis supports the utility of NSD‐ISS in defining the stages of disease progression, at least in the early clinical and prodromal stages (2B, 3, or 4), suggesting the value of NSD‐ISS as a potential research tool for drug development. Further research involving preclinical cohorts is a crucial next step. © 2025 The Author(s). *Movement Disorders* published by Wiley Periodicals LLC on behalf of International Parkinson and Movement Disorder Society.

Two research biological definitions of Parkinson's disease (PD) and related disorders have recently been proposed.^1^, ^2^ Neuronal α‐synuclein disease (NSD) is defined by the presence of an in vivo biomarker of neuronal alpha‐synuclein (n‐asyn) pathology.^1^ SynNeurGe is a three‐component system that is also anchored in the presence of n‐asyn biomarker (S) and biomarkers of neurodegeneration (N) but also includes individuals who are S biomarker negative and have relevant genetic variants (G).^2^ Both definitions encompass PD, dementia with Lewy bodies (DLB), and any other n‐asyn‐driven clinical syndrome. We have further proposed the NSD integrated staging system (NSD‐ISS) that integrates the biological substrates of the disease, n‐asyn (S) and dopaminergic dysfunction (D), with cognitive, other nonmotor, or motor manifestations and increasing degree of functional impairment to define stages along the NSD continuum in the research setting. This first iteration of the NSD‐ISS aims to provide an integrated biological and clinical research framework to facilitate research to expand our understanding of disease and advance biologically targeted therapeutic development.

The NSD‐ISS proposed seven distinct stages: stage 0 (presence of fully penetrant pathogenic variants in *SNCA* gene); stage 1 (presence of n‐asyn alone [stage 1A] or in combination with dopaminergic dysfunction [stage 1B], asymptomatic); stage 2 (presence of n‐asyn alone [stage 2A] or in combination with dopaminergic dysfunction [stage 2B], and subtle clinical signs/symptoms without functional impairment); and stages 3 to 6 (presence of both n‐asyn and dopaminergic dysfunction, and clinical signs/symptoms with progressively increasing severity of functional impairment).^1^ We have subsequently developed biologic and clinical criteria and thresholds, utilizing currently available clinical and functional rating scales, to operationalize the NSD and NSD‐ISS framework and have applied these definitions to assess cross‐sectional baseline staging utilizing available baseline data in three well‐characterized studies.^3^ These results demonstrated that staging by NSD‐ISS separated these early disease cohorts into NSD stages 2, 3, and 4, highlighting the significant heterogeneity in samples defined based on established clinical diagnostic criteria or features. The data further demonstrated that stage at baseline was a strong predictor of progression to clinically meaningful milestones.^3^ However, the longitudinal stability of the NSD‐ISS and the time for progression from one stage to the next have not been explored yet. These variables are a key requirement for a disease staging system. The aims of this analysis were to assess the (1) longitudinal change in the NSD‐ISS, (2) domains of functional impairment (motor, nonmotor, cognition) that lead to stage change, and (3) the impact of PD medications on the stability of stage.

## Patients and Methods

### Participants and Study Design

Data were obtained from the Parkinson's Progression Markers Initiative (PPMI) study. Study aims and methodology have been published elsewhere.^4^, ^5^, ^6^ Briefly, PPMI (NCT01141023) is a multinational, prospective, longitudinal, observational study launched in 2010.^4^ Initial enrollment consisted of participants with early PD and healthy controls (HC). Subsequently, the number of cohorts was increased, including participants with PD and nonmanifesting carriers of genetic variants associated with PD and participants with prodromal features (rapid eye movement sleep behavior disorder [RBD] or hyposmia). Individuals with PD were enrolled in the early PD cohort if they fulfilled PPMI inclusion criteria for PD cohort,^4^ were within 2 years of diagnosis, were in Hoehn and Yahr (HY) stages 1 to 2, were not on PD medications at the time of enrollment, and had an abnormal dopamine transporter (DAT) imaging scan with single‐photon emission computed tomography (SPECT). Inclusion criteria for the genetic PD cohort were the same, except that PD medications and diagnosis within 7 years were allowed. Prodromal participants exhibited prodromal features associated with risk of PD, including severe hyposmia as measured by the University of Pennsylvania Smell Identification Test based on internal population norms^7^ or RBD confirmed by a polysomnogram. HCs were similar in age and sex and individuals without known neurological signs or symptoms and normal DAT imaging. For this analysis, only participants enrolled before 2020 (allowing the opportunity for at least 5 years of follow‐up) who were n‐asyn positive were included.

All PPMI participants underwent extensive clinical phenotypic and biological characterization annually. Participants underwent a series of clinical assessments as described previously.^4^, ^5^, ^6^ Relevant assessments included the Montreal Cognitive Assessment^8^ and the Movement Disorders Society Unified Parkinson's Disease Rating Scale (MDS‐UPDRS), Parts I (nonmotor aspects or experiences of daily living), II (motor aspects or experiences of daily living), and III (motor examination; recorded in the *off* state at baseline for treated participants).^9^ Cerebrospinal fluid (CSF) samples were collected annually. DAT SPECT imaging was acquired longitudinally per protocol.^10^

All study and recruitment materials were approved by institutional review boards or ethics committees at each site. Written informed consent was obtained from all participants before undergoing any study evaluations. The study was performed in accordance with the principles outlined in the Declaration of Helsinki and with Good Clinical Practice guidelines.

### Eligibility and Staging

NSD stage was assigned to each annual visit for all PPMI participants enrolled prior to 2020 who were NSD positive and had sufficient data to determine stage. NSD status was determined based on the positive results of the CSF α‐synuclein seed amplification assay (αSyn‐SAA). All samples were handled and processed using standardized procedures and were analyzed at a central laboratory using standardized assay conditions (Amprion, San Diego, CA, USA). Each individual sample was analyzed in triplicate and determined to be either αSyn‐SAA positive (S+) or negative (S−) according to a previously reported algorithm.^11^, ^12^ Data on sensitivity and specificity of the assay have been previously published.^11^, ^13^, ^14^, ^15^ Participants were excluded if they had no CSF n‐asyn available or if they did not have at least one follow‐up visit. If participants attended a follow‐up but were missing necessary data to determine their stage, data from the previous annual visit were carried forward. As previously published, stages were assigned according to the change in DAT binding (over stages 2A to 2B), presence of nonmotor and motor symptoms (over stages 0–6), and level of functional impairment as determined by the components of MDS‐UPRDS, Parts 1 and 2 (stages 3–6)^3^ (Table S1).

### Statistical Analysis

NSD stage at baseline was tabulated for the overall sample and separately by enrollment cohort (PD, prodromal, HC) and subgroup (eg, sporadic vs. genetic PD). Descriptive statistics at baseline were presented overall and by stage at baseline, including frequency (percentage) for categorical measures and mean and standard deviation or median and interquartile range for continuous measures. χ^2^ or Fisher's exact tests for categorical measures and Kruskal–Wallis tests for continuous measures were used to compare baseline characteristics across NSD stages 2B to 4. A Bonferroni‐adjusted α‐level of 0.0025 was used to determine statistical significance.

Longitudinal analyses of change in stage were restricted to stage 2 and beyond due to a small sample size in stages 0 to 1B (n = 13) and excluded stage 2A participants who did not have at least one follow‐up DAT SPECT scan (n = 8). The frequency of the NSD stage over time was tabulated at each annual visit through year 5 for all observed data. Stage over time was additionally presented for the subset of participants who completed a year 5 visit, using the last stage carried forward if participants did not have data for an interim visit; Sankey diagrams depicting stage progression in those stages 2B to 4 at baseline among those who completed a year 5 visit were presented separately using the same rules. The frequency of the HY stage over time was tabulated using the same methods.

Kaplan–Meier plots were constructed for time from enrollment to reaching the first‐stage increase, stratified by baseline stage (for stages 2B–4). Median time to stage increase and 95% confidence intervals (CI) are presented for each group. Participants who did not increase in stage or were lost to follow‐up were right censored, with time to censoring calculated as the number of years from the enrollment date to the last follow‐up visit date. Among participants whose first‐stage increase was to stages 3 to 5, the tracks (ie, cognitive, motor, and/or other nonmotor) that contributed to the stage increase were tabulated by stage at first progression.

Kaplan–Meier plots were also constructed for the time from enrollment to initiation of symptomatic therapy, stratified by baseline stage (for stages 2A–4), and excluding participants on PD medication at baseline. Initiation of PD medication was defined as the first visit after a participant began actively taking PD medications (ie, any medication contributing to the levodopa equivalency dose calculation).^16^, ^17^ Median time to initiating medication and 95% CI are presented for each group. Participants who did not initiate medication or were lost to follow‐up were right censored, with time to censoring calculated as years from the enrollment date to the last follow‐up visit date.

Among those who initiated PD medication in stages 2B to 4 at the last visit prior to medication initiation, change in stage and key outcomes used to determine stage are presented for the last visit prior to initiating medication and the first visit on medication. Participants were excluded from this table if they started medication <90 days from the first visit on medication or if they could not be definitively staged at the visits of interest without carrying forward missing observations. The tracks contributing to stage at the last visit before initiation of PD medications were compared in stage reverters versus nonreverters among participants in stages 3 and 4 at the last visit prior to initiating PD medications.

Analyses were conducted using SAS, version 9.4 (SAS Institute Inc., Cary, NC; sas.com; RRID:SCR 008567). Sankey diagrams were prepared using “ggsankey” in R statistical software (version 4.3.2; R Core Team 2023).^18^

### Role of the Funding Source

Research officers (S.C., C.M.K., T.S., and Y.X.) at MJFF, PPMI Sponsor, were involved in the design of the study and writing of the manuscript.

## Results

A total of 576 participants were included in the analysis. See Figure S1 for the study flow chart and reasons for exclusion from the analysis. Table 1 provides baseline staging for the participants presented for the overall cohort and by enrollment cohort and subgroup. For the overall cohort, 56% of individuals were in Stage 3, defined by biomarkers of n‐asyn and dopamine dysfunction and slight functional impairment, which is consistent with the majority of participants enrolled as early PD based on PPMI enrollment criteria. There was substantial heterogeneity of staging at baseline in individuals recruited with the inclusion criteria of early sporadic PD (Table 1). Whereas the majority were in Stage 3, 24% were in stage 2B and 10% in stage 4. Participants recruited in the genetic PD subgroups, who were enrolled up to 7 years after diagnosis, had a higher percentage in stage 4. As expected, most individuals recruited into the RBD and hyposmia subgroups of the prodromal cohort were stage 2 (presence of clinical signs but no functional impairment), although 13% to 17% were in Stage 3. Only a minority of individuals recruited as nonmanifesting carriers of relevant genetic variants (*GBA, LRRK2, SNCA*) fulfilled NSD criteria (Fig. S1; Table 1). Among those, the majority were in stage 2A/2B. Eight HCs (4% of those evaluable) fulfilled NSD criteria, stages 1 and 2.

**TABLE 1:** Baseline staging of the PPMI NSD cohort

		PPMI enrollment cohort									
		PD (N = 494)				Prodromal (N = 74)					HC (N = 8)
Stage	All participants (N = 576)	Sporadic PD (N = 345)	*LRRK*2 PD* (N = 87)	*GBA* PD (N = 53)	*SNCA* PD (N = 9)	RBD (N = 30)	Hyposmia (N = 18)	*LRRK*2 NMC** (N = 15)	*GBA* NMC (N = 10)	*SNCA* NMC (N = 1)	HC (N = 8)
Stage 0	1 (<1%)	–	–	–	0 (NA)	–	–	–	–	1 (NA)	–
Stage 1A	10 (2%)	0	0	0	0 (NA)	0	0	4 (27%)	3 (30%)	0 (NA)	3 (NA)
Stage 1B	2 (<1%)	0	0	0	0 (NA)	0	0	2 (13%)	0	0 (NA)	0 (NA)
Stage 2A	24 (4%)	2 (1%)	0	0	0 (NA)	4 (13%)	4 (22%)	5 (33%)	6 (60%)	0 (NA)	3 (NA)
Stage 2B	137 (24%)	83 (24%)	12 (14%)	7 (13%)	1 (NA)	18 (60%)	11 (61%)	3 (20%)	0	0 (NA)	2 (NA)
Stage 3	324 (56%)	227 (66%)	53 (61%)	32 (60%)	3 (NA)	4 (13%)	3 (17%)	1 (7%)	1 (10%)	0 (NA)	0 (NA)
Stage 4	73 (13%)	33 (10%)	20 (23%)	13 (25%)	4 (NA)	3 (10%)	0	0	0	0 (NA)	0 (NA)
Stage 5	4 (1%)	0	1 (1%)	1 (2%)	1 (NA)	1 (3%)	0	0	0	0 (NA)	0 (NA)
Stage 6	1 (<1%)	0	1 (1%)	0	0 (NA)	0	0	0	0	0 (NA)	0 (NA)

Data shown as n (%); percentages shown for subgroups with ≥10 participants only.

* LRRK2 PD subgroup includes 3 participants who also carried a GBA mutation (2 in Stage 3, 1 in stage 4).

** LRRK2 NMC subgroup includes 2 participants who also carried a GBA mutation (1 in stage 1A, 1 in stage 2A).

Abbreviations: PPMI, Parkinson's Progression Markers Initiative; NSD, neuronal α‐synuclein disease; PD, Parkinson's disease; HC, healthy control; RBD, rapid eye movement sleep behavior disorder; NA, not applicable; NMC, nonmanifesting carriers.

Baseline demographic and clinical characteristics and biomarker data of the participants with baseline NSD‐ISS stages 2A to 4 are presented in Table S2a. Stages with fewer 20 participants were excluded from the main table and instead presented in Table S2b. Particularly, there was stage‐dependent reduction in the DAT SPECT mean striatum‐specific binding ratio; 33% of participants had CSF aβ 1 to 42 below the established cutoff though there was no stage‐dependent difference. There was no stage‐dependent separation of the other reported biomarkers after multiple comparisons were accounted for.

**FIG. 1:**
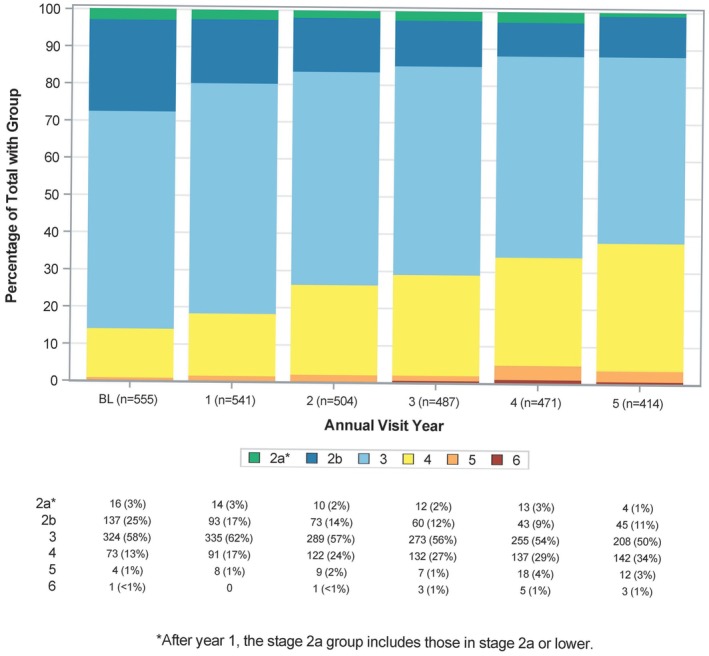
Longitudinal staging of the PPMI (Parkinson's Progression Markers Initiative) NSD cohort. Report generated on data submitted as of February 5, 2024. NSD, neuronal α‐synuclein disease; NSD‐ISS, neuronal α‐synuclein disease integrated staging system. *After year 1, stage 2A group includes those in stage 2A or lower. **Excludes participants who were in stage 0, 1A, or 1B at baseline (n = 13) or stage 2A at baseline without follow‐up assessment of dopaminergic dysfunction (n = 8).

Longitudinal staging of the cohort as a whole is shown in Figure 1. At the cohort level, there was a reduction in the percentage of individuals in stage 2B, a relatively stable percentage of individuals in Stage 3, and an increase in stage 4, reflecting expected progression across stages. A number of individuals did not progress across stages during the reported follow‐up. Sensitivity analysis on the 414 participants with observed 5‐year data yielded very similar results (Fig. S2). We assessed HY longitudinal staging of the same cohort (Fig. S3). Although both staging frameworks exhibited progression over 5 years, the majority of participants remained in HY stages 1 to 2, whereas a significant proportion of individuals progressed to NSD Stage 3 and beyond on NSD staging, indicating a new onset of functional impairment.

**FIG. 2:**
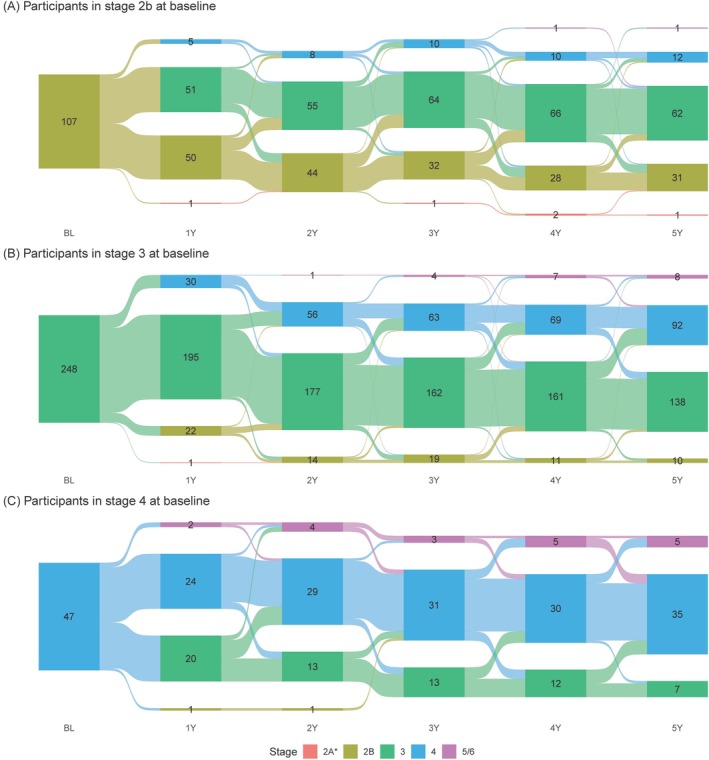
Participant flow. (**A**) Participants in stage 2b at baseline. (**B**) Participants in Stage 3 at baseline. (2**C**) Participants in stage 4 at baseline. The solid bars represent the number of participants in each stage category at each annual visit (BL, 1Y, 2Y, 3Y, 4Y, 5Y). The lighter‐shaded curved bands show the “flow” between stage categories from one year to the next. Participants were included if they competed the year 5 visit and were in stages 2B, 3, or 4 at baseline. In the case of intermittent missing annual visits, stage was carried forward.

**FIG. 3:**
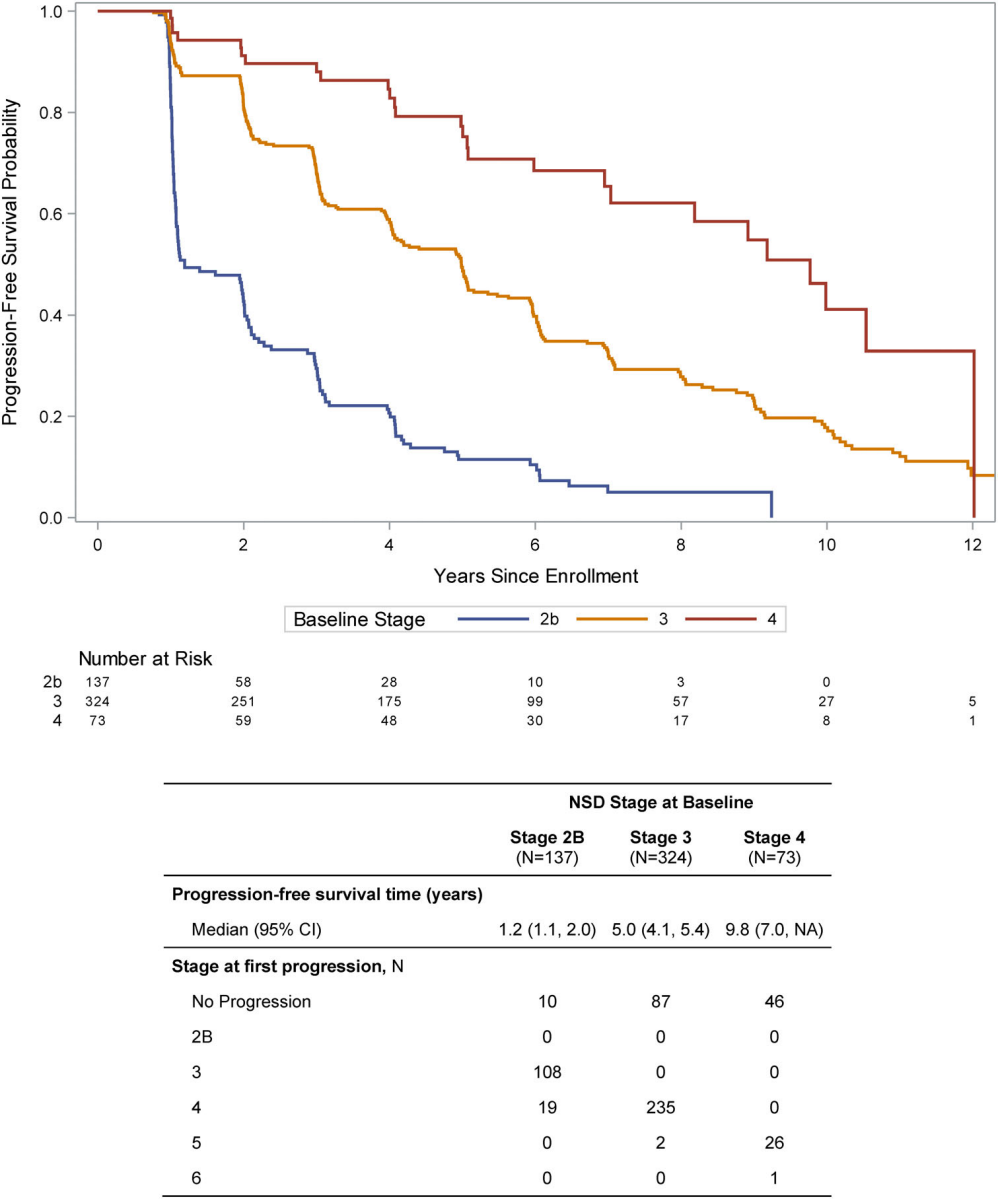
Time to reaching next‐stage increase by baseline stage.

We further explored stage progression at the participant level by including only 414 individuals with 5 years of observed data (Fig. 2). A majority of participants with baseline stage 2B progressed to Stage 3, with a few “reversions” back (Fig. 2A). A higher proportion of participants with baseline Stage 3 remained stable, and those who progressed did not revert (Fig. 2B). The majority of participants in baseline stage 4 remained in that stage, with a few advancing to stage 5/6 and a number reverting to Stage 3 (Fig. 2C). For the individuals who progressed, the majority advanced to the next stage, though 19 of 137 stage 2B progressors advanced to stage 4 (Fig. 3, bottom table).

Although these data do not allow us to determine the time from *onset* of each stage to progression to the next, we were able to examine the time to first‐stage progression from *baseline*. Figure 3 and the accompanying table present the data on the time for stage increase for participants across stages 2B to 4. Time to progression to the next stage increased with increasing stage. Median (95% CI) time to next‐stage increase from baseline was 1.2 (1.1–2.0) years for stage 2B, 5.0 (4.1–5.4) years for Stage 3, and 9.8 (7.0– not applicable (NA)) years for stage 4.

We also assessed the impact of baseline staging on time to initiation of PD medications (Fig. S4). The analysis was restricted to individuals who were not taking such at baseline. The median (95% CI) time to initiation of PD medications was 2.1 (2.0–3.0) years in stage 2B, 1.1 (1.1–1.2) years in Stage 3, and 1.1 (1.1–1.2) years in stage 4. We further assessed the impact of initiation of PD medications on stage stability and progression (Table S3). Importantly, there was no stage reversion (reduction) in individuals who were in stage 2B prior to PD medication initiation, and low number of reversions in baseline Stage 3 (8%), whereas there was significant reversion (41%) in baseline stage 4. The majority of individuals in stage 2B progressed in stage despite initiation of PD medications, whereas for the majority of individuals in baseline Stage 3, the stage remained stable (79%).

Considering that NSD‐ISS provides anchors for stage progression across motor, cognitive, and other nonmotor domains, we assessed what “track” led to stage progression (Table 2). The majority of individuals progressing to Stage 3 progressed only on the motor “track” (91%), whereas individuals progressing to stage 4 advanced based on all three tracks though still with a higher proportion on the motor track. Progression to stage 5 was driven by cognitive domain in 29%, motor domain in 36%, and nonmotor domain in 11%, with a notably low number advancing based on combination of tracks. We performed a similar analysis for the time to stage progression and tracks within 3 years from baseline as a time period more relevant for clinical trials (Table S4). Overall conclusions were similar. To further assess the impact of initiation of PD medications on stage stability or reversion, we assessed the “tracks” of progression in individuals whose stage remained stable or progressed versus reversed (Table S5A,B) and did not identify clear correlation with the motor track, though the number of reversions was small.

**TABLE 2:** Tracks leading to stage progression

Track	Subgroup		
	Progressed to Stage 3* (N = 110)	Progressed to stage 4** (N = 255)	Progressed to stage 5*** (N = 28)
Met criteria for domain^a^			
Cognitive	10 (9%)	40 (16%)	11 (39%)
Motor	105 (95%)	152 (60%)	17 (61%)
Other nonmotor	N/A	117 (46%)	8 (29%)
Combination of domains			
Cognitive only	5 (5%)	27 (11%)	8 (29%)
Motor only	100 (91%)	106 (42%)	10 (36%)
Other nonmotor only	N/A	73 (29%)	3 (11%)
Cognitive + motor	5 (5%)	5 (2%)	2 (7%)
Motor + other nonmotor	N/A	36 (14%)	4 (14%)
Cognitive + other nonmotor	N/A	3 (1%)	0
Cognitive + motor + other nonmotor	N/A	5 (2%)	1 (4%)

* At baseline, 108 participants were in stage 2B and 2 were in stage 2A.

** At baseline, 235 participants were in Stage 3, 19 were in stage 2B, and 1 was in stage 2A.

*** At baseline, 26 participants were in stage 4 and 2 were in Stage 3.

^a^ Indicates if criteria for the corresponding domain were met irrespective of whether criteria for other domains were also met.

## Discussion

We present the data on longitudinal change in the NSD‐ISS across the continuum of individuals with early PD and prodromal PD and HCs recruited in the PPMI study who fulfilled NSD criteria at baseline. We tracked stage changes in the PPMI cohort over 5 years of follow‐up. Most participants either remained at their baseline stage or progressed to the next higher stage during their observation period. Most individuals did not skip a stage or revert to a lower stage. Our focus was on stages 2B, 3, and 4 where the majority of these participants were at baseline. Notably, there was progressive reduction in the number of individuals in stage 2B and an increase in stage 4, whereas the numbers in Stage 3 remained relatively stable. This can be explained by the expected progression of stage 2B to 3 and Stage 3 to 4, supporting the concept of staging. Sensitivity analysis in individuals with observed 5‐year data demonstrated the same pattern. We recognize that there is an essential need to assess timelines and baseline predictors of progression in NSD stages 0 to 2A, but these cohorts are currently being recruited and longitudinal data are not available yet.

Hoehn and Yahr staging is a widely used clinical staging system in PD research and has its advantages, including that it can be easily applied based only on clinical evaluation.^19^ However, with the shift toward defining disease biologically, the NSD‐ISS integrates clinical/functional and biomarker measures of the disease. Our data indicate that this approach has value, given a broader representation of stages when applying the NSD‐ISS compared to the HY. Specifically, comparison of progression on NSD‐ISS versus HY staging demonstrated that although there was progression in both staging frameworks, most of the participants remained in HY stage 2 or below, whereas a significant proportion of individuals progressed to NSD Stage 3, indicative of new onset of functional impairment. This suggests that NSD‐ISS offers higher sensitivity to capture early but meaningful functional impairment in earlier stages, which importantly is not limited strictly to motor dysfunction.

Consistent with the previous report,^3^ we demonstrated significant baseline stage heterogeneity in individuals recruited into the study under the current definition of early PD. Such heterogeneity can have a significant impact on the readouts of disease‐modifying interventional studies as it adds to the variance of the cohort.

We also present data on baseline clinical and biological characteristics of participants in stages 0 to 6, though predominantly stages 2B to 4, demonstrating stage‐dependent separation of a number of clinical variables that are not part of the staging anchors. Importantly, we demonstrate stage‐dependent separation of DAT quantitative imaging characteristics even though DAT is applied as a binary measure (normal or abnormal) in the current version of NSD‐ISS. We did not observe a meaningful separation in the other tested biomarkers, but the panel was limited, highlighting the need to assess a wide spectrum of additional biological markers in stage‐dependent progression. Such work is underway, including a panel of biomarkers of neurodegeneration and inflammation.

Because the majority of these data are from the original PPMI cohort enrolled as early PD (not by stage), it is unclear how long these individuals had been in their stage at baseline. Therefore, although these data are useful in describing heterogeneity of the baseline staging, they do not allow us to accurately determine the timing of progression from the onset of one stage to the next for these participants. However, we have provided data for this cohort for the time from baseline to first‐stage progression. These data suggest that for this early PD and prodromal cohort, the change in early stages (stages 2B and 3) is within the time frame acceptable for the design of interventional studies. For example, 50% of individuals progressed from stage 2B to 3 by 1.2 (1.1, 2.0) years from recruitment and 75% by 3.1 (2.9, 4.1) years from recruitment. Such a time frame can make stage progression a viable outcome for disease modification studies once validated in other cohorts. These results should be interpreted with caution as individuals recruited into this cohort might represent “the tail end” of stage 2B. Further investigation in a cohort followed from the start of stage 2B is underway in PPMI.

The initiation of PD medications in clinical trials targeting early PD has been a key confounding factor in the interpretation of longitudinal data such as the MDS‐UPDRS. Most studies target individuals who do not take PD medications and censor the data once such medications were initiated. An alternative approach piloted recently is time to event analysis that has been demonstrated to be less susceptible to initiation of PD medications.^20^ Although we envision that the NSD‐ISS provides the framework to start interventional studies in earlier stages when PD medications are not relevant, our data provide a number of important observations supporting relative “resistance” of the NSD‐ISS, specifically in early stages (stages 2B and 3), to the effect of PD medications. Our data also provide another rationale to exclude individuals in stage 4 from disease‐modifying trials due to faster time to initiation of PD medications, higher dropout rate, and higher percentage of stage reversal after initiation of PD medications. However, it is expected that initiation of PD medications will lead to stage reversion in clinical stages that are anchored to the level of functional impairment, highlighting the ultimate need for quantitative biomarker–based staging. Future analyses will need to explore key factors predicting stage reversion.

In summary, the strength of our data is the comprehensive determination of stage progression in a large cohort of biologically defined and deeply phenotypically characterized individuals. The study has a number of limitations that serve to set the objectives for future research. Most importantly, the majority of the cohort with more than 5 years of longitudinal data were recruited as early PD and as expected fall predominantly into Stage 3. Although 24% of the cohort were in stage 2B, most of them still had a PD clinical phenotype (but no functional impairment). Less than 10% of the cohort were in stage 2A or below. As therapeutic development moves into disease prevention, the data on timelines and predictors of stage 1 and 2A progression are essential. Identification of stage 1 cohorts (biomarkers strictly defined) will require population screening and will be predicated on availability of feasible and scalable biomarkers. The field is actively working on this, and we hope that such cohorts will be launched in the next 2 to 5 years. Identification of stage 2A individuals is feasible with currently available tools, and the PPMI is aiming to recruit about 1000 such participants in the next 5 years. Similarly, we have very limited representation of individuals with advanced NSD stages 5 to 6. It is also important to recognize that the PPMI cohort is a research cohort, not representative of the PD general population, and as such baseline staging and longitudinal progression in real‐life cohorts may be different. We emphasize that the NSD‐ISS is a research framework not to be applied in the clinical setting. This analysis applied NSD staging to all qualified participants based on their baseline NSD status and staging. It is plausible that individuals with genetic variants and other subtypes might have a different NSD‐ISS progression trajectory, which will be a focus of future analyses. This analysis utilized NSD anchors developed by our group based on the data from PPMI and two recent interventional studies.^3^ These anchors need to be applied and validated in other cohorts, and very likely might be revised. This applies to the anchors and cutoffs for all domains but specifically for the cognitive one as these measures are highly dependent on cohort demographic and socioeconomic characteristics. Any revision of the anchors will lead to the change in NSD staging. We hope that this publication will stimulate the application and potential further revisions of the staging anchors. Another limitation is that the currently reported PPMI cohorts do not include individuals with DLB or prodromal DLB and have a limited number of individuals with stage 2B RBD phenotype. Since 2020, we have recruited a significant number of RBD stage 2A and 2B individuals and will be reporting longitudinal data as the cohort develops. Based on the conceptual framework of NSD, NSD staging can be applied only to individuals who fulfill the NSD diagnostic criteria, that is, have biomarker evidence of n‐asyn. Therefore, this analysis excluded two categories of individuals who were recruited with the clinical diagnosis of PD: (1) participants who were S‐, G‐ but had evidence of dopaminergic dysfunction (D+) and (2) participants who were S‐, D+ but G+ (a subset of *LRRK2*‐PD as an example). The first group comprises about 6% of the PPMI sporadic PD enrollment^12^ cohort and does not meet either NSD or SynNeurGe criteria. The latter group does fulfill SynNeurGe criteria. Both groups are essential for understanding the biological drives of PD and related disorders. Although they do not satisfy the NSD criteria, and as such cannot be staged using the NSD‐ISS, analysis of the baseline and longitudinal characteristics of both subgroups enrolled in the PPMI is underway, and the data will be published shortly.

In conclusion, we report data on the longitudinal change in the NSD‐ISS staging focusing on stages 2B to 4, provide data on the timeline of stage progression, and address a number of questions that are important to facilitate the implementation of the NSD‐ISS as a research tool for therapeutic development. We hope that these data will encourage the application of the NSD‐ISS in other cohorts, including cohorts with baseline nonmotor phenotypes to inform the field. Validation in interventional studies will be crucial. Further work involving preclinical cohorts is an essential next step.

## Author Roles

All authors contributed to conceptualization, investigations, methodology, and supervision. T.S., C.G., A.R.N., M.C.B., K.L.P., L.M.C., D.W., P.G.‐L., G.P., and A.S. contributed to review and editing of the final version of the manuscript. T.S., C.G., A.R.N., M.C.B., C.C., L.M.C., A.S., B.D., and K.M. contributed to data curation, formal analysis, and validation. T.S., C.G., A.R.N., M.C.B., C.C., L.M.C., D.W., P.G.‐L., T.D., G.P., A.S., and K.M. contributed to the writing of the original draft. T.S., C.M.K., Y.X., and S.C. contributed to project administration, acquired funding, and provided resources. All authors had direct access to and verified the underlying data.

## Funding information

Funding support for data analysis was provided by The Michael J. Fox Foundation for Parkinson's Research (MJFF). PPMI, a public–private partnership, is funded by MJFF and funding partners, including 4D Pharma, AbbVie, AcureX, Allergan, Amathus Therapeutics, Aligning Science Across Parkinson's, AskBio, Avid Radiopharmaceuticals, Bial, BioArctic, Biogen, Biohaven, BioLegend, BlueRock Therapeutics, BristolMyers Squibb, Calico Labs, Capsida Biotherapeutics, Celgene, Cerevel Therapeutics, Coave Therapeutics, DaCapo Brainscience, Denali, Edmond J. Safra Foundation, Eli Lilly, Gain Therapeutics, GE HealthCare, Genentech, GSK, Golub Capital, Handl Therapeutics, Insitro, Janssen Neuroscience, Jazz Pharmaceuticals, Lundbeck, Merck, Meso Scale Discovery, Mission Therapeutics, Neurocrine Biosciences, Neuropore, Pfizer, Piramal, Prevail Therapeutics, Roche, Sanofi, Servier, Sun Pharma Advanced Research Company, Takeda, Teva, UCB, Vanqua Bio, Verily, Voyager Therapeutics, the Weston Family Foundation, and Yumanity Therapeutics.

## Full financial disclosures of all authors for the previous 12 months

T.S. declares consultancy fees from AcureX, Adamas, AskBio, Amneal, Blue Rock Therapeutics, Critical Path for Parkinson's Consortium, Denali, The Michael J. Fox Foundation, Neuroderm, Roche, Sanofi, Sinopia, Takeda, and Vanqua Bio; advisory board fees from AcureX, Adamas, AskBio, Biohaven, Denali, GAIN, Neuron23, and Roche; scientific advisory board fees from Koneksa, Neuroderm, Sanofi, and UCB; and research funding from Amneal, Biogen, Roche, Neuroderm, Sanofi, Prevail, and UCB. T.S. is an investigator for NINDS, MJFF, and Parkinson's Foundation. C.G. declares research funding to her institution from The Michael J. Fox Foundation. A.R.N. declares research funding to her institution from The Michael J. Fox Foundation. M.C.B. declares travel grants from The Michael J. Fox Foundation. C.C. declares grants from The Michael J. Fox Foundation and NIH/NINDS. K.L.P. declares consultancy fees from Curasen; was on the scientific advisory board of Curasen and Amprion; received honoraria from invited scientific presentations to universities and professional societies not exceeding $5000 per year from the California Congress of Clinical Neurology, the California Neurological Society, and Johns Hopkins University; and has patents or patent application numbers 17/314,979 and 63/377,293. K.L.P. also declares grants to her institution (Stanford University School of Medicine) from NIH/NINDS NS115114, NS062684, NS075097, NIH/NIA U19 AG065156, P30 AG066515, The Michael J. Fox Foundation, Lewy Body Dementia Association, Alzheimer's Drug Discovery Foundation, and Sue Berghoff. L.M.C. declares grants to her institution from Biogen (clinical trial funding), MJFF, UPMC Competitive Medical Research Fund, the National Institutes of Health, and the University of Pittsburgh; grant and travel support from MJFF; royalties from Wolters Kluwer (for authorship); and in‐kind donation from Advanced Brain Monitoring of equipment for research study to her institution. D.W. declares salary support from The Michael J. Fox Foundation for serving on an executive steering committee for the PPMI and consultancies for Roche Pharma. In addition, he has received research funding or support from The Michael J. Fox Foundation for Parkinson's Research, the International Parkinson and Movement Disorder Society (IPMDS), the National Institute on Health (NIH), the Parkinson's Foundation, and the U.S. Department of Veterans Affairs; honoraria for consultancy from AbbVie, Boehringer Ingelheim, Cerevel Therapeutics, CHDI Foundation, Citrus Health Group, Medscape, Modality.AI, Sage Therapeutics, Scion NeuroStim, Signant Health, and Vanqua Bio; and license fee payments from the University of Pennsylvania for the QUIP and QUIP‐RS. C.M.T. declares consultancy fees from CNS Ratings, Australian Parkinson's Mission, Biogen, Evidera, Cadent (data safety monitoring board), Adamas (steering committee), Biogen (via the Parkinson Study Group steering committee), Praxis (via the International Parkinsons and Movement Disorder Society), Kyowa Kirin (advisory board), Lundbeck (advisory board), Jazz/Cavion (steering committee), Acorda (advisory board), Bial (DMC), and Genentech. C.M.T. also declares grant support to her institution from The Michael J. Fox Foundation, the National Institutes of Health, Gateway LLC, Department of Defense, Roche Genentech, Biogen, Parkinson Foundation, and Marcus Program in Precision Medicine. C.M.T. declares membership on the *npj Parkinson's Disease* editorial board. P.G.‐L. decares an early investigator award from The Michael J. Fox Foundation. C.M.K. declares employment at The Michael J. Fox Foundation. Y.X. declares employment at and travel grants from The Michael J. Fox Foundation. S.C. declares employment at and travel grants from The Michael J. Fox Foundation. T.D. declares former employment at and employee stock options in Biogen. G.P. declares employment at F. Hoffmann‐La Roche Ltd. and stock ownership for F. Hoffmann‐La Roche Ltd., Atea, Novartis, and Eli Lilly. D.S. has nothing to declare. A.S. declares consultancy fees from Mitsubishi, GE HealthCare, Capsida Therapeutics, and Parkinson Study Group; grants from The Michael J. Fox Foundation (member of the PPMI Steering Committee); and fees for participation on DSMB boards at Spark Therapeutics, Cerevance, Alerity, Wave Life Sciences, Inhibikase, Prevai (Eli Lilly), the Huntington Study Group, and Massachusetts General Hospital. B.D. has received consulting fees and travel support for his role as an advisor for Arch Venture Partners, Cerveau Technologies, Epilepsy Foundation, Loulou Foundation, and the Michael J Fox Foundation. B.D. has a leadership or fiduciary role in the Virginia Neurological Society (past president) and Prothena (Director). B.D. holds stock options with Prothena. K.M. declares support to his institution (Institute for Neurodegenerative Disorders) from The Michael J. Fox Foundation. K.M. also declares consultancy fees from Invicro, The Michael J. Fox Foundation, Roche, Calico, Coave, Neuron23, Orbimed, Biohaven, Anofi, Koneksa, Merck, Lilly, Inhibikase, Neuramedy, IRLabs, and Prothena and participates on DSMB at Biohaven.

## Supporting information


**Figure S1.** Study flowchart.
**Figure S2.** Longitudinal staging of the PPMI (Parkinson's Progression Markers Initiative) NSD (neuronal α‐synuclein disease) cohort (completers only last observation carried forward (LOCF)).
**Figure S3.** Longitudinal Hoehn and Yahr (*on*) staging of the PPMI (Parkinson's Progression Markers Initiative) NSD (neuronal α‐synuclein disease) cohort.
**Figure S4.** Time to initiation of PD (Parkinson's disease) medication by baseline stage (excluding those on medication at baseline).
**Table S1.** Staging anchors for application of the NSD‐ISS (neuronal α‐synuclein disease‐integrated staging system).
**Table S2A.** Clinical and biological baseline characteristics of the participants.
**Table S2B.** Clinical and biological baseline characteristics—omitted groups.
**Table S3.** Impact of PD (Parkinson's disease) medications on NSD (neuronal α‐synuclein disease) stage and key outcomes at “last *off* medications visit” versus “first *on* medications visit” among participants who initiated symptomatic therapy (ST).
**Table S4.** Tracks leading to stage progression (within 3 years).
**Table S5.** A and B. Tracks leading defining stage at last *off* PD (Parkinson's disease) medication visit in stage reverters versus nonreverters.

## Data Availability

Data used in the preparation of this article were obtained on February 5, 2024, from the Parkinson's Progression Markers Initiative (PPMI) database (www.ppmi-info.org/access-data-specimens/download-data), RRID:SCR_006431. Up‐to‐date information on the study is available at www.ppmi-info.org. Protocol information for the Parkinson's Progression Markers Initiative (PPMI) Clinical—Establishing a Deeply Phenotyped PD Cohort AM 3.2. is available at protocols.io or at https://doi.org/10.17504/protocols.io.n92ldmw6ol5b/v2. This analysis was conducted by the PPMI Statistics Core and used actual dates of activity for participants, a restricted data element not available to public users of PPMI data. Statistical analysis codes used to perform the analyses in this article are shared on Zenodo, https://doi.org/10.5281/zenodo.14889347 (Appendix A).

## Appendix A PPMI STUDY TEAMS/CORES/COLLABORATORS FOR PUBLICATIONS

**Executive Steering Committee:**

Kenneth Marek, MD^1^; Caroline Tanner, MD, PhD^9^; Tanya Simuni, MD^3^; Andrew Siderowf, MD, MSCE^12^; Douglas Galasko, MD^27^; Lana Chahine, MD^39^; Christopher Coffey, PhD^4^; Kalpana Merchant, PhD^59^; Kathleen Poston, MD^38^; Roseanne Dobkin, PhD^41^; Tatiana Foroud, PhD^15^; Brit Mollenhauer, MD^8^; Dan Weintraub, MD^12^; Ethan Brown, MD^9^; Karl Kieburtz, MD, MPH^23^; Mark Frasier, PhD^6^; Todd Sherer, PhD^6^; Sohini Chowdhury, MA^6^; Roy Alcalay, MD^35^; and Aleksandar Videnovic, MD^45^

**Steering Committee:**

Duygu Tosun‐Turgut, PhD^9^; Werner Poewe, MD^7^; Susan Bressman, MD^14^; Jan Hammer^15^; Raymond James, RN^22^; Ekemini Riley, PhD^40^; John Seibyl, MD^1^; Leslie Shaw, PhD^12^; David Standaert, MD, PhD^18^; Sneha Mantri, MD, MS^60^; Nabila Dahodwala, MD^12^; Michael Schwarzschild^45^; Connie Marras^43^; Hubert Fernandez, MD^25^; Ira Shoulson, MD^23^; Helen Rowbotham^2^; Paola Casalin^11^ and Claudia Trenkwalder, MD^8^

**The Michael J. Fox Foundation (Sponsor):** Todd Sherer, PhD; Sohini Chowdhury, MA; Mark Frasier, PhD; Jamie Eberling, PhD; Katie Kopil, PhD; Alyssa O'Grady; Maggie McGuire Kuhl; Leslie Kirsch, EdD, and Tawny Willson, MBS

**Study Cores, Committees, and Related Studies:**

*Project management core*: Emily Flagg, BA^1^

*Site management core*: Tanya Simuni, MD^3^; Bridget McMahon, BS^1^

Strategy and technical operations: Craig Stanley, PhD^1^; Kim Fabrizio, BA^1^

*Data management core*: Dixie Ecklund, MBA, MSN^4^; Trevis Huff, BSE^4^

*Screening core*: Tatiana Foroud, PhD^15^; Laura Heathers, BA^15^; Christopher Hobbick, BSCE^15^; Gena Antonopoulos, BSN^15^

*Imaging core*: John Seibyl, MD^1^; Kathleen Poston, MD^38^

*Statistics core*: Christopher Coffey, PhD^4^; Chelsea Caspell‐Garcia, MS^4^ ; Michael Brumm, MS^4^

*Bioinformatics core*: Arthur Toga, PhD^10^; Karen Crawford, MLIS^10^ *Biorepository core*: Tatiana Foroud, PhD^15^; Jan Hamer, BS^15^

*Biologics review committee*: Brit Mollenhauer^8^; Doug Galasko^27^; Kalpana Merchant^59^

*Genetics core*: Andrew Singleton, PhD^13^

*Pathology core*: Tatiana Foroud, PhD^15^; Thomas Montine, MD, PhD^38^

*Found*: Caroline Tanner, MD PhD^9^

*PPMI Online*: Carlie Tanner, MD PhD^9^; Ethan Brown, MD^9^; Lana Chahine, MD^39^ Roseann Dobkin, PhD^41^; Monica Korell, MPH^9^

**Site Investigators:**

Charles Adler, PhD^49^; Roy Alcalay, MD^35^; Amy Amara, PhD^50^; Paolo Barone, PhD^30^; Bastiaan Bloem, PhD^58^; Susan Bressman, MD^14^; Kathrin Brockmann, MD^26^; Norbert Brüggemann, MD^57^; Lana Chahine, MD^39^; Kelvin Chou, MD^42^; Nabila Dahodwala, MD^12^; Alberto Espay, MD^32^; Stewart Factor, DO^16^; Hubert Fernandez, MD^25^; Michelle Fullard, MD^50^; Douglas Galasko, MD^27^; Robert Hauser, MD^19^; Penelope Hogarth, MD^17^; Shu‐Ching Hu, PhD^21^; Michele Hu, PhD^56^; Stuart Isaacson, MD^31^; Christine Klein, MD^57^; Rejko Krueger, MD^2^; Mark Lew, MD^47^; Zoltan Mari, MD^54^; Connie Marras, PhD^43^; Maria Jose Martí, PhD^33^; Nikolaus McFarland, PhD^52^; Tiago Mestre, PhD^44^; Brit Mollenhauer, MD^8^; Emile Moukheiber, MD^28^; Alastair Noyce, PhD^61^; Wolfgang Oertel, PhD^62^; Njideka Okubadejo, MD^63^; Sarah O’Shea, MD^37^; Rajesh Pahwa, MD^46^; Nicola Pavese, PhD^55^; Werner Poewe, MD^7^; Ron Postuma, MD^53^; Giulietta Riboldi, MD^51^; Lauren Ruffrage, MS^18^; Javier Ruiz Martinez, PhD^34^; David Russell, PhD^1^; Marie H Saint‐Hilaire, MD^22^; Neil Santos, BS^49^; Wesley Schlett^45^; Ruth Schneider, MD^23^; Holly Shill, MD^48^; David Shprecher, DO^24^; Tanya Simuni, MD^3^; David Standaert, PhD^18^; Leonidas Stefanis, PhD^36^; Yen Tai, PhD^29^; Caroline Tanner, PhD^9^; Arjun Tarakad, MD^20^; Eduardo Tolosa PhD^33^ and Aleksandar Videnovic, MD^45^

**Coordinators:**

Susan Ainscough, BA^30^; Courtney Blair, MA^18^; Erica Botting^19^; Isabella Chung, BS^54^; Kelly Clark^24^; Ioana Croitoru^34^; Kelly DeLano, MS^32^; Iris Egner, PhD^7^; Fahrial Esha, BS^51^; May Eshel, MSc^35^; Frank Ferrari, BS^42^; Victoria Kate Foster^55^; Alicia Garrido, MD^33^; Madita Grümmer^57^; Bethzaida Herrera^48^; Ella Hilt^26^; Chloe Huntzinger, BA^50^; Raymond James, BS^22^; Farah Kausar, PhD^9^; Christos Koros, MD, PhD^36^; Yara Krasowski, MSc^58^; Dustin Le, BS^17^; Ying Liu, MD^50^; Taina M. Marques, PhD^2^; Helen Mejia Santana, MA^37^; Sherri Mosovsky, MPH^39^; Jennifer Mule, BS^25^; Philip Ng, BS^43^; Lauren O’Brien^46^; Abiola Ogunleye, PGDip^29^; Oluwadamilola Ojo, MD^63^; Obi Onyinanya, BS^28^; Lisbeth Pennente, BA^31^; Romina Perrotti^53^; Michael Pileggi, MS^53^; Ashwini Ramachandran, MSc^12^; Deborah Raymond, MS^14^; Jamil Razzaque, MS^56^; Shawna Reddie, BA^44^; Kori Ribb, BSN^28^; Kyle Rizer, BA^52^; Janelle Rodriguez, BS^27^; Stephanie Roman, HS^1^; Clarissa Sanchez, MPH^20^; Cristina Simonet, PhD^29^; Anisha Singh, BS^23^; Elisabeth Sittig, RN^62^; Barbara Sommerfeld MSN^16^; Angela Stovall, BS^42^; Bobbie Stubbeman, BS^32^; Alejandra Valenzuela, BS^47^; Catherine Wandell, BS^21^; Diana Willeke^8^; Karen Williams, BA^3^ and Dilinuer Wubuli, MB^43^

**Partners Scientific Advisory Board (Acknowledgment)**

Funding: PPMI, a public–private partnership, is funded by The Michael J. Fox Foundation for Parkinson's Research and funding partners, including 4D Pharma, AbbVie, AcureX, Allergan, Amathus Therapeutics, Aligning Science Across Parkinson's, AskBio, Avid Radiopharmaceuticals, Bial, BioArctic, Biogen, Biohaven, BioLegend, BlueRock Therapeutics, Bristol‐Myers Squibb, Calico Labs, Capsida Biotherapeutics, Celgene, Cerevel Therapeutics, Coave Therapeutics, DaCapo Brainscience, Denali, Edmond J. Safra Foundation, Eli Lilly, Gain Therapeutics, GE HealthCare, Genentech, GSK, Golub Capital, Handl Therapeutics, Insitro, Jazz Pharmaceuticals, Johnson & Johnson Innovative Medicine, Lundbeck, Merck, Meso Scale Discovery, Mission Therapeutics, Neurocrine Biosciences, Neuron23, Neuropore, Pfizer, Piramal, Prevail Therapeutics, Roche, Sanofi, Servier, Sun Pharma Advanced Research Company, Takeda, Teva, UCB, Vanqua Bio, Verily, Voyager Therapeutics, the Weston Family Foundation, and Yumanity Therapeutics.

^1^Institute for Neurodegenerative Disorders, New Haven, CT, USA

^2^University of Luxembourg, Luxembourg

^3^Northwestern University, Chicago, IL, USA

^4^University of Iowa, Iowa City, IA, USA

^5^VectivBio AG, Basel, Switzerland

^6^The Michael J. Fox Foundation for Parkinson's Research, New York, NY, USA

^7^Innsbruck Medical University, Innsbruck, Austria

^8^Paracelsus‐Elena Klinik, Kassel, Germany

^9^University of California, San Francisco, CA, USA

^10^Laboratory of Neuroimaging (LONI), University of Southern California, Los Angeles, CA, USA

^11^BioRep, Milan, Italy

^12^University of Pennsylvania, Philadelphia, PA, USA

^13^National Institute on Aging, NIH, Bethesda, MD, USA

^14^Mount Sinai Beth Israel, New York, NY, USA

^15^Indiana University, Indianapolis, IN, USA

^16^Emory University of Medicine, Atlanta, GA, USA

^17^Oregon Health and Science University, Portland, OR, USA

^18^University of Alabama at Birmingham, Birmingham, AL, USA

^19^University of South Florida, Tampa, FL, USA

^20^Baylor College of Medicine, Houston, TX, USA

^21^University of Washington, Seattle, WA, USA

^22^Boston University, Boston, MA, USA

^23^University of Rochester, Rochester, NY, USA

^24^Banner Research Institute, Sun City, AZ, USA

^25^Cleveland Clinic, Cleveland, OH, USA

^26^University of Tübingen, Tübingen, Germany

^27^University of California, San Diego, CA, USA

^28^Johns Hopkins University, Baltimore, MD, USA

^29^Imperial College of London, London, United Kingdom

^30^University of Salerno, Salerno, Italy

^31^Parkinson's Disease and Movement Disorders Center, Boca Raton, FL, USA

^32^University of Cincinnati, Cincinnati, OH, USA

^33^Hospital Clinic of Barcelona, Barcelona, Spain

^34^Hospital Universitario Donostia, San Sebastian, Spain

^35^Tel Aviv Sourasky Medical Center, Tel Aviv, Israel

^36^National and Kapodistrian University of Athens, Athens, Greece

^37^Columbia University Irving Medical Center, New York, NY, USA

^38^Stanford University, Stanford, CA, USA

^39^University of Pittsburgh, Pittsburgh, PA, USA

^40^Center for Strategy Philanthropy at Milken Institute, Washington, DC, USA

^41^Rutgers University, Robert Wood Johnson Medical School, New Brunswick, NJ, USA

^42^University of Michigan, Ann Arbor, MI, USA

^43^Toronto Western Hospital, Toronto, ON, Canada

^44^The Ottawa Hospital, Ottawa, ON, Canada

^45^Massachusetts General Hospital, Boston, MA, USA

^46^University of Kansas Medical Center, Kansas City, KS, USA

^47^University of Southern California, Los Angeles, CA, USA

^48^Barrow Neurological Institute, Phoenix, AZ, USA

^49^Mayo Clinic Arizona, Scottsdale, AZ, USA

^50^University of Colorado, Aurora, CO, USA

^51^NYU Langone Medical Center, New York, NY, USA

^52^University of Florida, Gainesville, FL, USA

^53^Montreal Neurological Institute and Hospital/McGill, Montreal, QC, Canada

^54^Cleveland Clinic‐Las Vegas Lou Ruvo Center for Brain Health, Las Vegas, NV, USA

^55^Clinical Ageing Research Unit, Newcastle, United Kingdom

^56^John Radcliffe Hospital Oxford and Oxford University, Oxford, United Kingdom

^57^Universität Lübeck, Luebeck, Germany

^58^Radboud University, Nijmegen, the Netherlands

^59^TransThera Consulting, Nanjing, China

^60^Duke University, Durham, NC, USA

^61^Wolfson Institute of Population Health, Queen Mary University of London, United Kingdom

^62^Philipps‐University Marburg, Marburg, Germany

^63^University of Lagos, Lagos, Nigeria

## References

[1] Simuni T , Chahine LM , Poston K , et al. A biological definition of neuronal alpha‐synuclein disease: towards an integrated staging system for research. Lancet Neurol 2024;23:178–190. 38267190 10.1016/S1474-4422(23)00405-2

[2] Hoglinger GU , Adler CH , Berg D , et al. A biological classification of Parkinson's disease: the SynNeurGe research diagnostic criteria. Lancet Neurol 2024;23:191–204. 38267191 10.1016/S1474-4422(23)00404-0

[3] Dam TP , Pagano G , Brumm M , et al. Neuronal alpha‐synuclein disease integrated staging system performance in PPMI, PASADENA, and SPARK baseline cohorts. Nature Partners Journal 2024;10(1):178. 10.1038/s41531-024-00789-w PMC11567150 39333167

[4] Marek K , Chowdhury S , Siderowf A , et al. The Parkinson's progression markers initiative (PPMI) ‐ establishing a PD biomarker cohort. Ann Clin Transl Neurol 2018;5:1460–1477. 30564614 10.1002/acn3.644 PMC6292383

[5] Pagano G , Taylor KI , Anzures‐Cabrera J , et al. Trial of Prasinezumab in early‐stage Parkinson's disease. N Engl J Med 2022;387:421–432. 35921451 10.1056/NEJMoa2202867

[6] Lang AE , Siderowf AD , Macklin EA , et al. Trial of Cinpanemab in early Parkinson's disease. N Engl J Med 2022;387:408–420. 35921450 10.1056/NEJMoa2203395

[7] Brumm MC , Pierz KA , Lafontant DE , et al. Updated percentiles for the University of Pennsylvania Smell Identification Test in adults 50 years of age and older. Neurology 2023;100:e1691–e1701. 36849448 10.1212/WNL.0000000000207077 PMC10115503

[8] Nasreddine ZS , Phillips NA , Bédirian V , et al. The Montreal cognitive assessment, MoCA: a brief screening tool for mild cognitive impairment. J Am Geriatr Soc 2005;53:695–699. 15817019 10.1111/j.1532-5415.2005.53221.x

[9] Goetz CG , Tilley BC , Shaftman SR , et al. Movement Disorder Society‐sponsored revision of the unified Parkinson's disease rating scale (MDS‐UPDRS): scale presentation and clinimetric testing results. Mov Disord 2008;23:2129–2170. 19025984 10.1002/mds.22340

[10] Marek K . The Parkinson's Progression Markers Initiative (PPMI) Clinical ‐ Establishing a Deeply Phenotyped PD Cohort AM 3.2. protocols.io; 2024. 10.17504/protocols.io.n92ldmw6ol5b/v2.

[11] Concha‐Marambio L , Farris CM , Holguin B , et al. Seed amplification assay to diagnose early Parkinson's and predict dopaminergic deficit progression. Mov Disord 2021;36:2444–2446. 34236720 10.1002/mds.28715 PMC8530949

[12] Siderowf A , Concha‐Marambio L , Lafontant DE , et al. Assessment of heterogeneity among participants in the Parkinson's progression markers initiative cohort using alpha‐synuclein seed amplification: a cross‐sectional study. Lancet Neurol 2023;22:407–417. 37059509 10.1016/S1474-4422(23)00109-6 PMC10627170

[13] Concha‐Marambio L , Pritzkow S , Shahnawaz M , Farris CM , Soto C . Seed amplification assay for the detection of pathologic alpha‐synuclein aggregates in cerebrospinal fluid. Nat Protoc 2023;18:1179–1196. 36653527 10.1038/s41596-022-00787-3 PMC10561622

[14] Concha‐Marambio L , Wang F , Armijo E , et al. Development of a methodology for large‐scale production of prions for biological and structural studies. Front Mol Biosci 2023;10:1184029. 37635939 10.3389/fmolb.2023.1184029 PMC10449461

[15] Concha‐Marambio L , Weber S , Farris CM , et al. Accurate detection of alpha‐synuclein seeds in cerebrospinal fluid from isolated rapid eye movement sleep behavior disorder and patients with Parkinson's disease in the DeNovo Parkinson (DeNoPa) cohort. Mov Disord 2023;38:567–578. 36781413 10.1002/mds.29329 PMC10153075

[16] Jost ST , Kaldenbach MA , Antonini A , et al. Levodopa dose equivalency in Parkinson's disease: updated systematic review and proposals. Mov Disord 2023;38:1236–1252. 37147135 10.1002/mds.29410

[17] Tomlinson CL , Stowe R , Patel S , et al. Systematic review of levodopa dose equivalency reporting in Parkinson's disease. Mov Disord 2010;25:2649–2653. 21069833 10.1002/mds.23429

[18] Joberg D . ggsankey: Sankey, Alluvial and Sankey Bump Plots. R package version 0.0.99999; https://github.com/davidsjoberg/ggsankey 2024.

[19] Cardoso F , Goetz CG , Mestre TA , et al. A Statement of the MDS on biological definition, staging, and classification of Parkinson's disease. Mov Disord 2024;39:259–266. 38093469 10.1002/mds.29683

[20] Brumm MC , Siderowf A , Simuni T , et al. Parkinson's progression markers initiative: a milestone‐based strategy to monitor Parkinson's disease progression. J Parkinsons Dis 2023;13:899–916. 37458046 10.3233/JPD-223433 PMC10578214

