# Supplementary material for: Neuronal α‐Synuclein Disease Stage Progression over 5 Years
Source: Mov Disord. 2025 Apr 30;40(7):1318–30. doi: 10.1002/mds.30191 (PMC12273616; doi:10.1002/mds.30191)
Supplement: Supplementary file 1 — Figure S1. Study flowchart. Figure S2. Longitudinal staging of the PPMI (Parkinson's Progression Markers Initiative) NSD (neuronal α‐synuclein disease) cohort (completers only last observation carried forward (LOCF)). Figure S3. Longitudinal Hoehn and Yahr (on) staging of the PPMI (Parkinson's Progression Markers Initiative) NSD (neuronal α‐synuclein disease) cohort. Figure S4. Time to initiation of PD (Parkinson's disease) medication by baseline stage (excluding those on medication at baseline). Table S1. Staging anchors for application of the NSD‐ISS (neuronal α‐synuclein disease‐integrated staging system). Table S2A. Clinical and biological baseline characteristics of the participants. Table S2B. Clinical and biological baseline characteristics—omitted groups. Table S3. Impact of PD (Parkinson's disease) medications on NSD (neuronal α‐synuclein disease) stage and key outcomes at “last off medications visit” versus “first on medications visit” among participants who initiated symptomatic therapy (ST). Table S4. Tracks leading to stage progression (within 3 years). Table S5. A and B. Tracks leading defining stage at last off PD (Parkinson's disease) medication visit in stage reverters versus nonreverters. [file MDS-40-1318-s001.pdf]

SUPPLEMENTARY MATERIAL

Supplementary Table 1. Staging anchors for application of the NSD-ISS

	Biologic anchors			Anchors of clinical signs or symptoms (stages 2A and 2B) and functional impairment (stages 3-6)[1, 2]	
**Stage**	**S**	**D** [a]	**G**	**Domain**	**Anchor(s)**
Stage 0	-	-	*SNCA* [b]	—	—
Stage 1A	+	-	±	(1) Cognitive<br>(2) Motor<br>(3) Other non-motor	(1) MDS-UPDRS item 1.1 = 0; and<br>(2a) Does not have subthreshold parkinsonism [c]; and (2b) is not on PD medication [d]; and<br>(3a) Does not have RBD; and (3b) is not hyposmic [e]
Stage 1B	+	+	±		
Stage 2A	+	-	±	(1) Cognitive<br>(2) Motor<br>(3) Other non-motor	(1) Item 1.1 = 1 AND MoCA ≥ 25; or<br>(2a) Has subthreshold parkinsonism [c]; or (2b) is on PD medication [d]; or<br>(3a) Has RBD; or (3b) is hyposmic [e]
Stage 2B	+	+	±		
Stage 3	+	+	±	(1) Cognitive<br>(2) Motor	(1a) Item 1.1 = 1 AND MoCA ≤ 24; or (1b) Item 1.1 = 2 AND MoCA ≥ 25; or<br>(2) MDS-UPDRS-II = 3-13 AND either subthreshold parkinsonism [c] or PD medication [d]
Stage 4	+	+	±	(1) Cognitive<br>(2) Motor<br>(3) Other non-motor	(1a) Item 1.1 = 2 and MoCA ≤ 24; or (1b) item 1.1 = 3 AND MoCA ≥ 25; or<br>(2) MDS-UPDRS-II = 14-26; or<br>(3) MDS-UPDRS-I (excluding item 1.1) = 13-24 [f]
Stage 5	+	+	±	(1) Cognitive<br>(2) Motor<br>(3) Other non-motor	(1a) Item 1.1 = 3 AND MoCA ≤ 24; or (1b) item 1.1 = 4 AND MoCA ≥ 25; or<br>(2) MDS-UPDRS-II = 27-39; or<br>(3) MDS-UPDRS-I (excluding item 1.1) = 25-36
Stage 6	+	+	±	(1) Cognitive<br>(2) Motor<br>(3) Other non-motor	(1) Item 1.1 = 4 AND MoCA ≤ 24; or<br>(2) MDS-UPDRS-II ≥ 40; or<br>(3) MDS-UPDRS-I (excluding item 1.1) ≥ 37

[1] Presence of qualifying signs/ symptoms in any single domain qualifies for stage 2 but individuals can have combination in all 3 domains.
[2] Presence of qualifying functional impairment in any single domain qualifies for stage 3-6 but individuals can have combination in all 3 domains.
[a] D positivity defined as < 75% age/sex-expected lowest putamen SBR.
[b] Only fully penetrant pathogenic *SNCA* variants qualify for Stage 0.
[c] Subthreshold parkinsonism defined as MDS-UPDRS-III ≥ 5 excluding postural and action tremor.
[d] Medication for treating the symptoms of PD as per MDS-UPDRS item 3a
[e] Hyposmia defined as UPSIT percentile ≤ 15 (age and sex adjusted).
[f] MDS-UPDRS-I (excluding item 1.1) ≥ 13 is sufficient for stage 4 provided that stage 2 criteria are met.

Supplementary Figure 1. Study flowchart

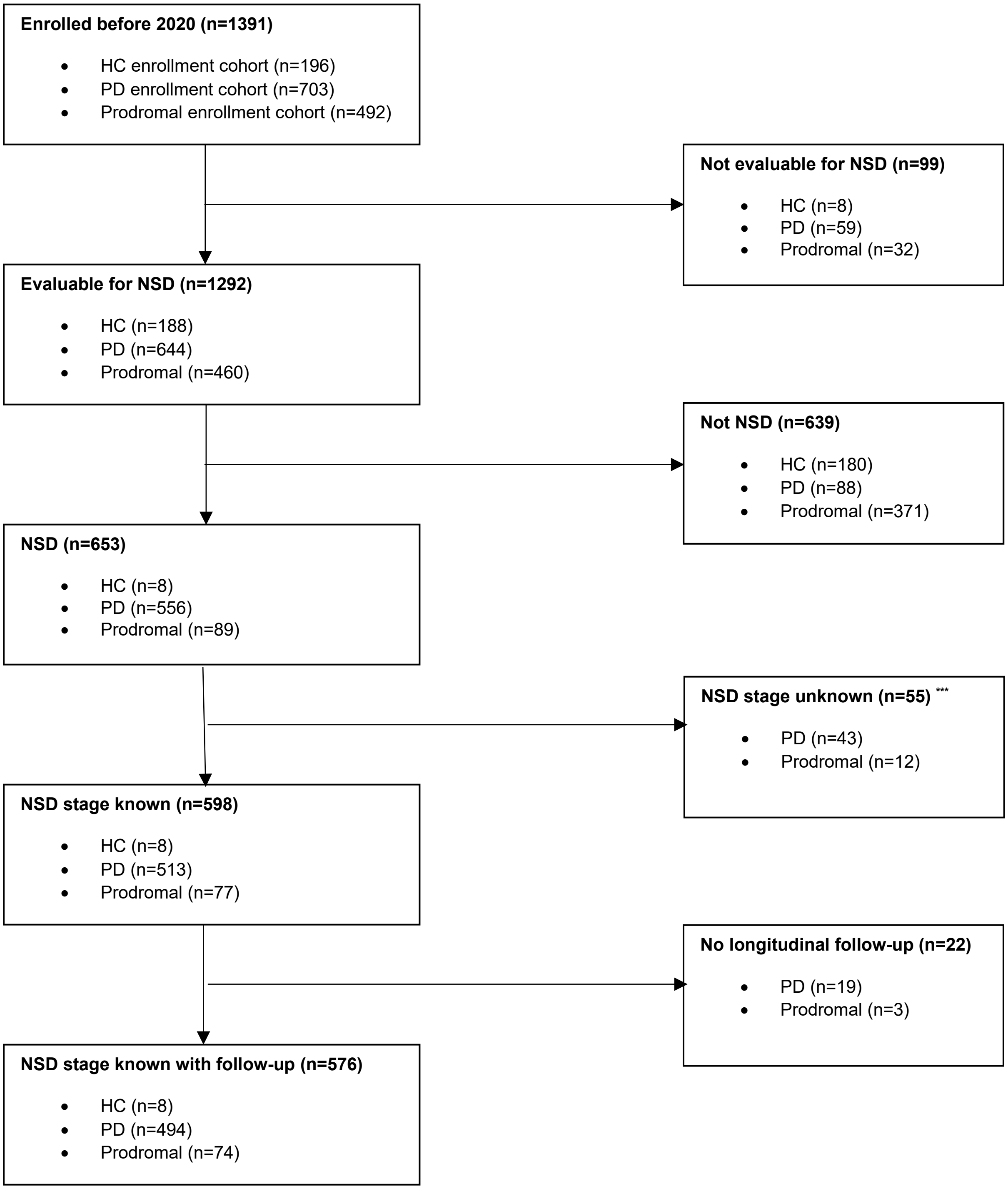

*HC, PD and Prodromal indicate PPMI enrollment cohorts
** Abbreviations: HC= Healthy Controls; NSD= neuronal alpha-synuclein disease; PD= Parkinsons's disease
Not evaluable = without CSF samples for alpha-synuclein aggregation testing and without an *SNCA* variant.
Evaluable = CSF samples analyzed for alpha-synuclein aggregation and/or carrier of an *SNCA* variant.
Stage unknown = missing data required to determine stage (e.g., DaT-SPECT or clinical data).
*** Out of the 43 PD with NSD stage unknown, 21 were missing DAT, 19 had SNCA variant but were missing SAA, 2 were missing Item 1.1 from MDS-UPDRS Part I scores, and 1 was missing MDS-UPDRS Part II score. Out of the 12 Prodromal with NSD stage unknown, 8 had SNCA variant but were missing SAA and 4 were missing DAT, hence, stage cannot be determined for these participants.

Supplemental Table 2a. Clinical and biological baseline characteristics of the participants

		NSD Stage at Baseline				
	**All Participants** (N = 576)	**Stage 2A** (N = 24)	**Stage 2B** (N = 137)	**Stage 3** (N = 324)	**Stage 4** (N = 73)	**P-value****
**Age (years),** Mean (SD)	61.8 (9.6)	65.7 (6.4)	62.7 (9.3)	61.8 (9.4)	59.1 (10.9)	0.02
**Sex (male),** n (%)	368 (64%)	17 (71%)	89 (65%)	211 (65%)	41 (56%)	0.446
**Years from PD dx,** Median (Q1, Q3)	0.5 (0.2, 1.6)	0.2 (0.2, 0.3)	0.4 (0.2, 0.9)	0.4 (0.2, 1.3)	1.7 (0.6, 3.1)	< 0.001
**MDS-UPDRS Item 1.1 Score,** Median (Q1, Q3)	0.0 (0.0, 1.0)	0.0 (0.0, 0.5)	0.0 (0.0, 0.0)	0.0 (0.0, 1.0)	0.0 (0.0, 1.0)	< 0.001
**MDS-UPDRS Part I,** Median (Q1, Q3)	5.0 (3.0, 9.0)	4.0 (1.5, 7.0)	3.0 (1.0, 6.0)	5.0 (3.0, 8.0)	14.0 (11.0, 16.0)	< 0.001
**MDS-UPDRS Part II,** Median (Q1, Q3)	5.0 (2.0, 8.0)	0.0 (0.0, 3.0)	2.0 (1.0, 2.0)	6.0 (4.0, 8.0)	14.0 (9.0, 15.0)	< 0.001
**MDS-UPDRS Part III (ON),** Median (Q1, Q3)	17.0 (10.0, 24.0)	0.0 (0.0, 4.0)	12.0 (5.5, 16.0)	20.0 (15.0, 25.0)	21.5 (13.5, 28.5)	< 0.001
Missing	6	0	1	4	1	
**MDS-UPDRS Total Score (ON),** Median (Q1, Q3)	28.0 (19.0, 39.0)	7.0 (2.0, 13.0)	17.0 (11.0, 22.5)	31.0 (25.0, 39.0)	47.0 (38.5, 55.0)	< 0.001
Missing	6	0	1	4	1	
**On PD Medication*,** n (%)	131 (23%)	0	18 (13%)	74 (23%)	35 (48%)	< 0.001
**Total LED,** Median (Q1, Q3)	500.0 (300.0, 830.0)	NA	300.0 (100.0, 415.0)	500.0 (300.0, 750.0)	700.0 (421.2, 905.0)	< 0.001
**MOCA Total Score,** Mean (SD)	26.9 (2.7)	27.5 (2.2)	27.2 (2.8)	26.9 (2.3)	26.5 (2.9)	0.117
Missing	2	0	0	0	1	
**UPSIT Percentile ≤ 15%,** n (%)	458 (80%)	20 (83%)	116 (87%)	253 (79%)	63 (88%)	0.132
Missing	7	0	3	3	1	
**Mean Striatum Binding,** Mean (SD)	1.46 (0.50)	2.56 (0.53)	1.51 (0.33)	1.36 (0.37)	1.21 (0.46)	< 0.001
**Age/Sex-Expected DAT,** Median (Q1, Q3)	0.33 (0.25, 0.43)	0.98 (0.85, 1.21)	0.37 (0.29, 0.48)	0.30 (0.25, 0.38)	0.26 (0.20, 0.36)	< 0.001
**Low CSF A-beta 1-42 (<683 pg/mL),** n (%)	187 (33%)	7 (29%)	38 (29%)	104 (33%)	33 (46%)	0.081
Missing	10	0	4	4	1	
**High CSF t-tau (>266 pg/mL),** n (%)	38 (7%)	1 (4%)	11 (8%)	18 (6%)	5 (7%)	0.721
Missing	6	0	3	2	0	
**High CSF p-tau (>24 pg/mL),** n (%)	32 (6%)	0	8 (6%)	19 (6%)	5 (7%)	0.758
Missing	6	0	3	2	0	
**Serum NFL,** Median (Q1, Q3)	11.7 (8.6, 16.0)	12.8 (10.1, 15.9)	12.3 (8.6, 16.8)	11.4 (8.6, 15.6)	12.0 (8.9, 17.4)	0.526
Missing	53	5	11	26	8	
**Serum Urate,** Mean (SD)	313.0 (80.6)	317.0 (83.6)	323.7 (81.8)	315.0 (79.6)	287.7 (77.4)	0.027
Missing	24	2	2	15	3	
**No. APOE e4 alleles,** n (%)						0.175
0	430 (75%)	13 (54%)	106 (77%)	242 (75%)	57 (79%)	
1	132 (23%)	10 (42%)	27 (20%)	76 (24%)	14 (19%)	
2	11 (2%)	1 (4%)	4 (3%)	5 (2%)	1 (1%)	
Missing	3	0	0	1	1	

Excludes participants who are in NSD stages 0, 1A, 1B, 5, and 6 at baseline with <20 participants in each group. Baseline characteristics for these groups are shown in Supplementary Table 2b.

*Based on enrollment criteria, genetic cohorts were allowed to be on PD medication at baseline.

**P-value tests for differences across stages 2A - 4. Chi-square or Fisher's exact tests were used for categorical variables and Kruskal-Wallis tests were used for continuous variables. We used a Bonferroni-adjusted α-level of 0.0025 to determine statistical significance.

Supplementary Table 2b. Clinical and Biological Baseline Characteristics - Omitted Groups

		NSD Stage at Baseline				
	**All Participants** (N = 576)	**Stage 0** (N = 1)	**Stage 1a** (N = 10)	**Stage 1b** (N = 2)	**Stage 5** (N = 4)	**Stage 6** (N = 1)
**Age (years),** Mean (SD)	61.8 (9.6)	44.8 (NA)	64.0 (8.7)	67.3 (NA)	58.7 (10.6)	49.6 (NA)
**Sex (male),** n (%)	368 (64%)	0 (NA)	5 (50%)	2 (NA)	3 (NA)	0 (NA)
**Years from PD dx,** Median (Q1, Q3)	0.5 (0.2, 1.6)	NA	NA	NA	2.1 (1.8, 5.9)	4.0 (NA)
Missing	82	1	10	2	1	0
**MDS-UPDRS Item 1.1 Score,** Median (Q1, Q3)	0.0 (0.0, 1.0)	0.0 (NA)	0.0 (0.0, 0.0)	0.0 (NA)	3.0 (2.0, 3.0)	0.0 (NA)
**MDS-UPDRS Part I,** Median (Q1, Q3)	5.0 (3.0, 9.0)	2.0 (NA)	3.5 (0.0, 6.0)	3.5 (NA)	15.0 (11.5, 18.5)	17.0 (NA)
**MDS-UPDRS Part II,** Median (Q1, Q3)	5.0 (2.0, 8.0)	0.0 (NA)	0.0 (0.0, 1.0)	1.0 (NA)	9.5 (3.5, 22.0)	40.0 (NA)
**MDS-UPDRS Part III (ON),** Median (Q1, Q3)	17.0 (10.0, 24.0)	0.0 (NA)	0.5 (0.0, 2.0)	0.0 (NA)	11.5 (7.5, 22.5)	16.0 (NA)
Missing	6	0	0	0	0	0
**MDS-UPDRS Total Score (ON),** Median (Q1, Q3)	28.0 (19.0, 39.0)	2.0 (NA)	5.0 (3.0, 7.0)	4.5 (NA)	43.0 (24.5, 61.0)	73.0 (NA)
Missing	6	0	0	0	0	0
**On PD Medication*,** n (%)	131 (23%)	0 (NA)	0	0 (NA)	3 (NA)	1 (NA)
**Total LED,** Median (Q1, Q3)	500.0 (300.0, 830.0)	NA	NA	NA	940.0 (750.0, 1190)	692.0 (NA)
**MOCA Total Score,** Mean (SD)	26.9 (2.7)	28.0 (NA)	27.4 (3.5)	27.5 (NA)	19.0 (5.9)	NA
Missing	2	0	0	0	0	1
**UPSIT Percentile ≤ 15%,** n (%)	458 (80%)	1 (NA)	0	0	4 (NA)	1 (NA)
Missing	7	0	0	0	0	0
**Mean Striatum Binding,** Mean (SD)	1.46 (0.50)	3.08 (NA)	2.88 (0.38)	1.56 (NA)	1.23 (0.23)	0.84 (NA)
**Age/Sex-Expected DAT,** Median (Q1, Q3)	0.33 (0.25, 0.43)	1.25 (NA)	1.11 (1.01, 1.23)	0.57 (NA)	0.37 (0.26, 0.44)	0.15 (NA)
**Low CSF A-beta 1-42 (<683 pg/mL),** n (%)	187 (33%)	0 (NA)	3 (30%)	1 (NA)	1 (NA)	NA
Missing	10	0	0	0	0	1
**High CSF t-tau (>266 pg/mL),** n (%)	38 (7%)	0 (NA)	0	1 (NA)	2 (NA)	NA
Missing	6	0	0	0	0	1
**High CSF p-tau (>24 pg/mL),** n (%)	32 (6%)	0 (NA)	0	0	0	NA
Missing	6	0	0	0	0	1
**Serum NFL,** Median (Q1, Q3)	11.7 (8.6, 16.0)	5.4 (NA)	9.2 (8.9, 10.8)	16.8 (NA)	22.3 (11.4, 40.6)	31.6 (NA)
Missing	53	0	2	0	1	0
**Serum Urate,** Mean (SD)	313.0 (80.6)	193.0 (NA)	298.3 (66.1)	387.0 (NA)	285.3 (50.2)	124.0 (NA)
Missing	24	0	2	0	0	0
**No. APOE e4 alleles,** n (%)						
0	430 (75%)	1 (NA)	7 (78%)	1 (NA)	2 (NA)	1 (NA)
1	132 (23%)	0	2 (22%)	1 (NA)	2 (NA)	0
2	11 (2%)	0	0	0	0	0
Missing	3	0	1	0	0	0

*Based on enrollment rules, genetic cohorts were allowed to be on PD medication at baseline.

Supplementary Figure 2. Longitudinal staging of the PPMI NSD cohort (completers only LOCF)

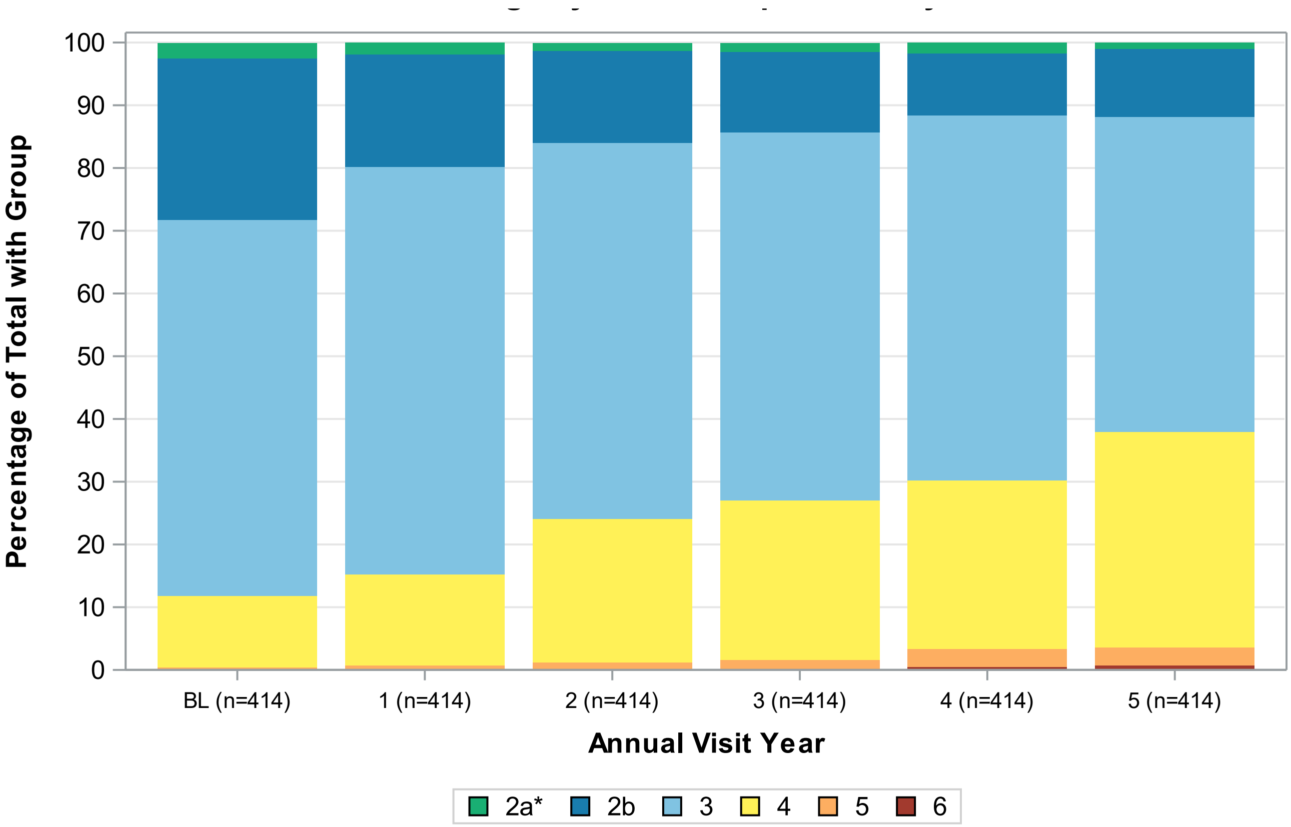

2a*	10 (2%)	8 (2%)	5 (1%)	6 (1%)	7 (2%)	4 (1%)
2b	107 (26%)	74 (18%)	61 (15%)	53 (13%)	41 (10%)	45 (11%)
3	248 (60%)	269 (65%)	248 (60%)	243 (59%)	241 (58%)	208 (50%)
4	47 (11%)	60 (14%)	95 (23%)	105 (25%)	111 (27%)	142 (34%)
5	1 (<1%)	3 (1%)	5 (1%)	6 (1%)	12 (3%)	12 (3%)
6	1 (<1%)	0	0	1 (<1%)	2 (<1%)	3 (1%)

*After year 1, the stage 2a group includes those in stage 2a or lower.

Abbreviations: NSD = neuronal alpha-synuclein disease; NSD-ISS = neuronal alpha-synuclein disease integrated staging system.
* After Year 1, Stage 2A group includes those in Stage 2A or lower.
** Excludes participants who are Stage 0, 1A and 1B (N=13) at baseline; participants who are Stage 2A at Baseline and have no follow up DAT (N=8). Limited to participants who competed the year 5 visit. In the case of intermittent missing annual visits, stage was carried forward.

Supplementary Figure 3. Longitudinal Hoehn and Yahr (ON) staging of the PPMI NSD cohort

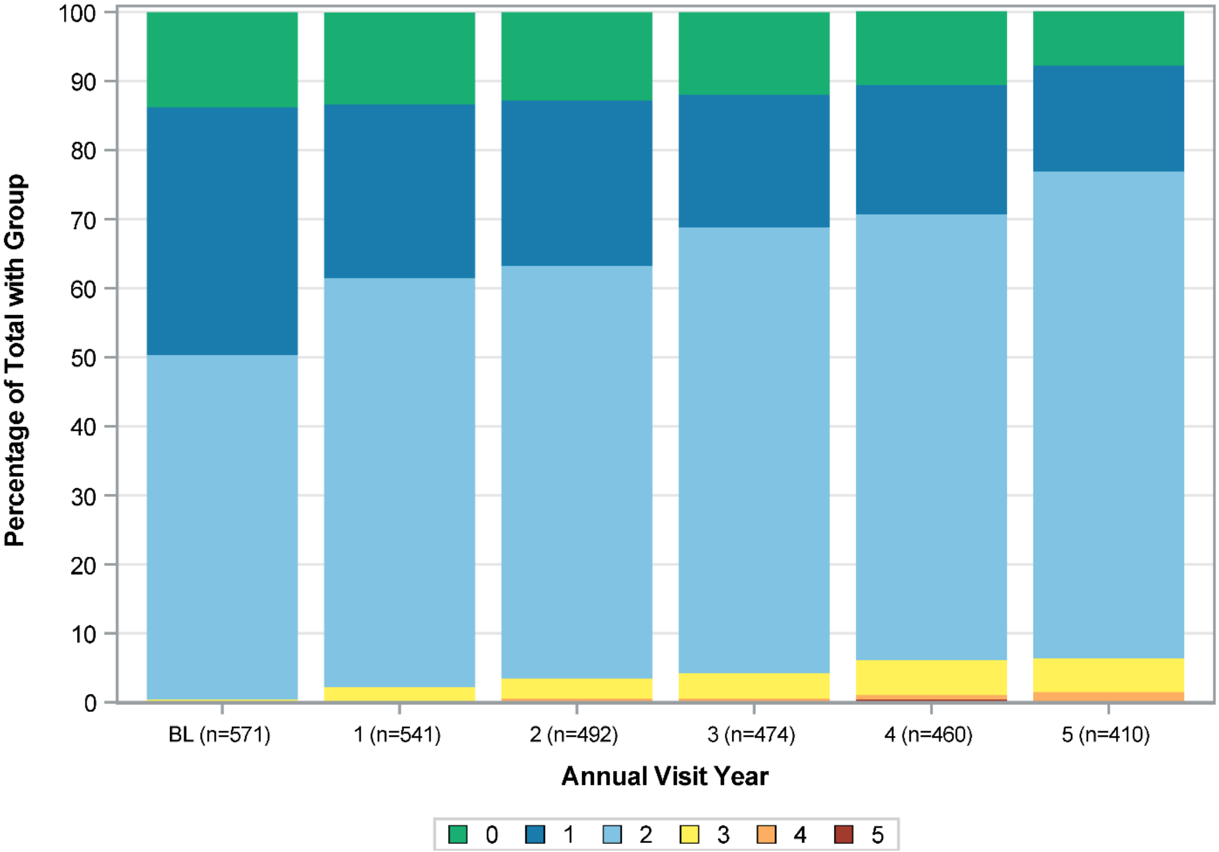

0	79 (14%)	72 (13%)	63 (13%)	57 (12%)	49 (11%)	32 (8%)
1	205 (36%)	136 (25%)	118 (24%)	91 (19%)	86 (19%)	63 (15%)
2	285 (50%)	321 (59%)	294 (60%)	306 (65%)	297 (65%)	289 (70%)
3	2 (<1%)	11 (2%)	14 (3%)	17 (4%)	23 (5%)	20 (5%)
4	0	1 (<1%)	3 (1%)	3 (1%)	3 (1%)	6 (1%)
5	0	0	0	0	2 (<1%)	0

Supplementary Table 3. Impact of PD medications on NSD stage and key outcomes at "last OFF medications visit" vs. "first ON medications visit" among participants who initiated ST *

Variable	Stage at last OFF visit<br>Stage 2b (N = 56)	 Stage 3 (N = 236)	 Stage 4 (N = 49)
**Stage status at first ON visit**, n (%)			
Stable	20 (36%)	187 (79%)	26 (53%)
Progressed	36 (64%)	30 (13%)	3 (6%)
Reverted	0	19 (8%)	20 (41%)
**MDS-UPDRS Item 1.1 at last OFF visit**, Mean (SD)	0.1 (0.3)	0.3 (0.5)	0.6 (0.8)
**MDS-UPDRS Item 1.1 at first ON visit**, Mean (SD)	0.3 (0.5)	0.4 (0.6)	0.7 (0.8)
**MDS-UPDRS I subscore at last OFF visit**, Mean (SD	2.9 (2.2)	5.4 (3.0)	12.0 (4.4)
**MDS-UPDRS I subscore at first ON visit**, Mean (SD	4.6 (2.9)	6.4 (3.6)	11.1 (5.4)
**MDS-UPDRS II at last OFF visit**, Mean (SD)	1.6 (0.7)	7.0 (3.0)	14.5 (3.8)
**MDS-UPDRS II at first ON visit**, Mean (SD)	4.5 (3.6)	7.5 (4.1)	13.0 (6.3)
**MDS-UPDRS III at last OFF visit**, Mean (SD)	16.3 (9.4)	24.5 (9.5)	31.4 (10.9)
Missing	0	2	0
**MDS-UPDRS III (ON) at first ON visit**, Mean (SD)	18.1 (11.0)	22.1 (10.4)	27.2 (12.7)
Missing	4	17	1
**MoCA at last OFF visit**, Mean (SD)	27.5 (2.0)	26.7 (2.5)	26.5 (2.8)
Missing	0	3	0
**MoCA at first ON visit**, Mean (SD)	26.9 (2.5)	26.4 (3.0)	25.8 (3.8)
**Total LED**, Mean (SD)	246.2 (152.4)	296.3 (213.1)	370.6 (239.1)
Missing	2	2	2

* Note: the "first ON visit" was defined as the first visit at which a participant was actively taking symptomatic therapy (i.e., any medication contributing to LED calculation).

Excludes n=29 participants who started LED medication <90 days from the first ON visit and n=5 participants who cannot be definitively staged without carrying forward missing observations.

Supplementary Figure 4. Time to initiation of PD medication by baseline stage (excluding those on medication at baseline)

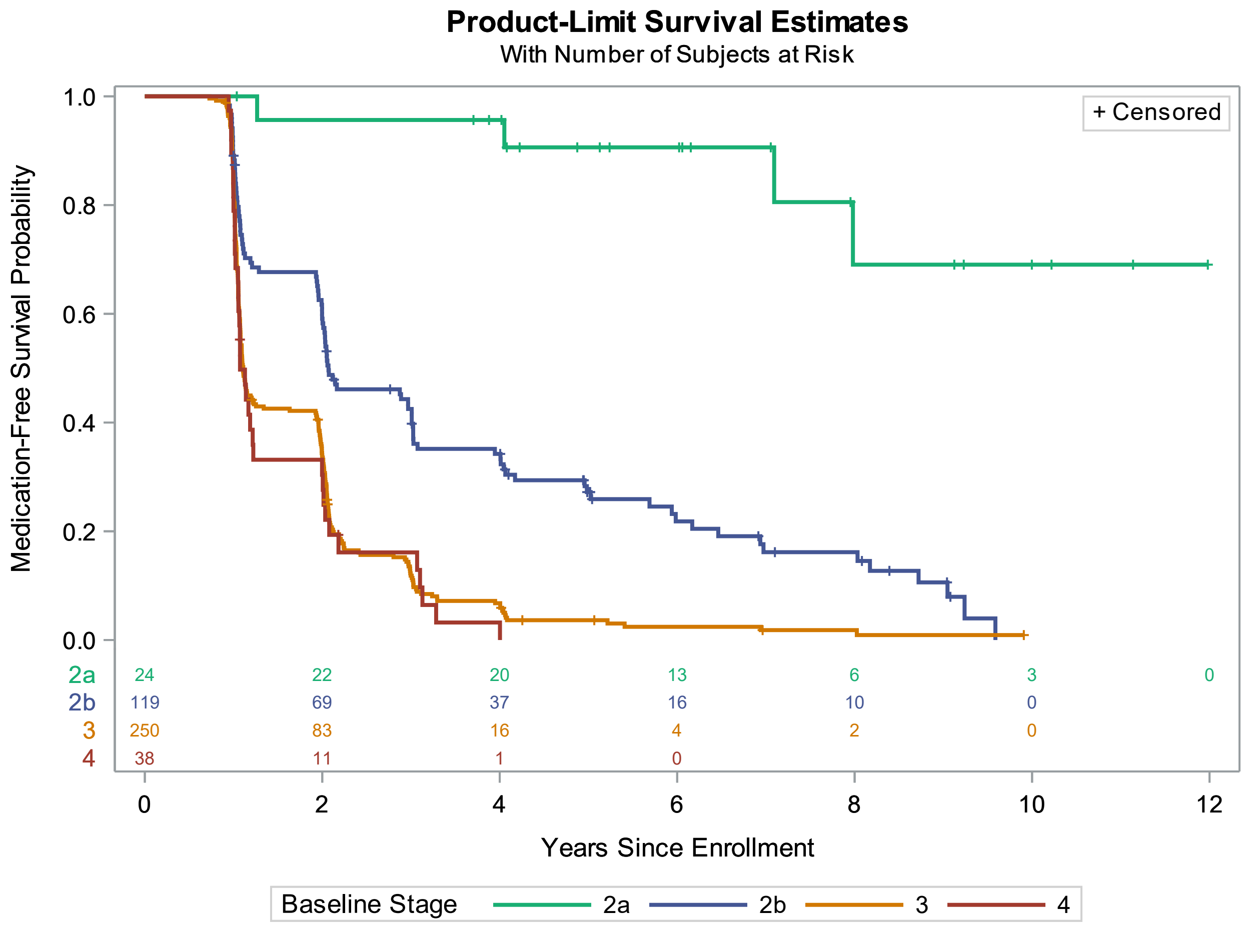

Baseline Stage	N	Initiated Medication	Censored	Median Years to Initiation of Medication (95% Confidence Interval)
2A	24	4	20	NA (7.09, NA)
2B	119	97	22	2.07 (2.00, 3.01)
3	250	240	10	1.11 (1.08, 1.22)
4	38	36	2	1.07 (1.05, 1.22)

Supplementary Table 4. Tracks leading to stage progression (within 3 years).

	Subgroup		
**Track**	**Progressed to Stage 3** [a] (N=92)	**Progressed to Stage 4** [b] (N=137)	**Progressed to Stage 5** [c] (N=11)
**Years to first progression**, mean (SD)	1.5 (0.8)	1.9 (0.8)	1.7 (0.8)
Median (IQR)	1.1 (1.0, 2.0)	2.0 (1.0, 3.0)	2.0 (1.0, 2.0)
**Met criteria for track**			
Cognitive	9 (10%)	28 (20%)	6 (55%)
Motor	87 (95%)	75 (55%)	4 (36%)
Non-Motor	N/A	69 (50%)	2 (18%)
**Combination of tracks**			
Cognitive only	5 (5%)	18 (13%)	6 (55%)
Motor only	83 (90%)	47 (34%)	3 (27%)
Other Non-Motor only	N/A	42 (31%)	1 (9%)
Cognitive + Motor	4 (4%)	3 (2%)	N/A
Motor + Other Non-Motor	N/A	20 (15%)	1 (9%)
Cognitive + Other Non-Motor	N/A	2 (1%)	N/A
Cognitive + Motor + Other Non-Motor	N/A	5 (4%)	N/A

a Reflects participants whose first progression was to stage 3. In most cases, these are individuals who started in stage 2B and progressed to stage 3.
b Reflects participants whose first progression was to stage 4. In most cases, these are individuals who started in stage 3 and progressed to stage 4.
c Reflects participants whose first progression was to stage 5. In most cases, these are individuals who started in stage 4 and progressed to stage 5.

Supplementary Table 5. Tracks defining stage at last OFF PD medications visit in stage reverters vs non-reverters

Table 5A. Analysis restricted to individuals who were stage 3 at last visit before initiation of PD medications

Track	Subgroup	
	**Stage remained stable or progressed** (N = 217)	**Stage Reverted** (N = 19)
**Met criteria for this domain(irrespective of other domains**, N(%)		
Cognitive	21 (10%)	1 (5%)
Motor	215 (99%)	18 (95%)
Non-Motor	N/A	N/A
**Met criteria for this domain exclusively**, N(%)		
Cognitive	2 (1%)	1 (5%)
Motor	196 (90%)	18 (95%)
Non-Motor	N/A	N/A
Cognitive + Motor	19 (9%)	N/A
Motor + Non-Motor	N/A	N/A
Cognitive + Non-Motor	N/A	N/A
Cognitive+ Motor+ Non-Motor	N/A	N/A

Table 5B. Analysis restricted to individuals who were stage 4 at last visit before initiation of PD medications

Track	Subgroup	
	**Stage remained stable or progressed** (N = 29)	**Stage reverted** (N = 20)
**Met criteria for this domain(irrespective of other domains**, N(%)		
Cognitive	2 (7%)	2 (10%)
Motor	19 (66%)	15 (75%)
Non-Motor	16 (55%)	7 (35%)
**Met criteria for this domain exclusively**, N(%)		
Cognitive	N/A	2 (10%)
Motor	13 (45%)	11 (55%)
Non-Motor	10 (34%)	3 (15%)
Cognitive + Motor	N/A	N/A
Motor + Non-Motor	4 (14%)	4 (20%)
Cognitive + Non-Motor	N/A	N/A
Cognitive+ Motor+ Non-Motor	2 (7%)	N/A
